# Polymeric resins containing modified starch as environmentally friendly adsorbents for dyes and metal ions removal from wastewater

**DOI:** 10.3389/fchem.2024.1496901

**Published:** 2024-10-30

**Authors:** Anna Wołowicz, Monika Wawrzkiewicz, Beata Podkościelna, Bogdan Tarasiuk, Jadranka Blazhevska Gilev, Olena Sevastyanova

**Affiliations:** ^1^ Faculty of Chemistry, Institute of Chemical Sciences, Department of Inorganic Chemistry, Maria Curie-Sklodowska University in Lublin, Lublin, Poland; ^2^ Faculty of Chemistry, Institute of Chemical Sciences, Department of Polymer Chemistry, Maria Curie-Sklodowska University in Lublin, Lublin, Poland; ^3^ Faculty of Technology and Metallurgy, Ss. Cyril and Methodius University in Skopje, Skopje, North Macedonia; ^4^ Wallenberg Wood Science Center, Department of Fibre and Polymer Technology, KTH Royal Institute of Technology, Stockholm, Sweden

**Keywords:** basic blue 3, acid green 16, heavy metals, removal, starch, polymeric adsorbents, wastewaters

## Abstract

Effective removal of organic and inorganic impurities by adsorption technique requires the preparation of new materials characterized by low production costs, significant sorption capacity, and reduced toxicity, derived from natural and renewable sources. To address these challenges, new adsorbents have been developed in the form of polymer microspheres based on ethylene glycol dimethacrylate (EGDMA) and vinyl acetate (VA) (EGDMA/VA) containing starch (St) modified with boric acid (B) and dodecyl-S-thiuronium dodecylthioacetate (DiTDTA) for the removal of dyes: C.I. Basic Blue 3 (BB3) and C.I. Acid Green 16 (AG16) and heavy metal ions (M(II)): Cu(II), Ni(II), and Zn(II) from water and wastewater. The adsorbents were characterized by ATR/FT-IR, DSC, SEM, BET, EDS, and pH_PZC_ methods. These analyses demonstrated the successful modification of microspheres and the increased thermal resistance resulting from the addition of the modified starch. The point of zero charge for EGDMA/VA was 7.75, and this value decreased with the addition of modified starch (pH_PZC_ = 6.62 for EGDMA/VA-St/B and pH_PZC_ = 5.42 for EGDMA/VA-St/DiTDTA). The largest specific surface areas (S_BET_) were observed for the EGDMA/VA microspheres (207 m^2^/g), and S_BET_ value slightly decreases with the modified starch addition (184 and 169 m^2^/g) as a consquence of the pores stopping by the big starch molecules. The total pore volumes (V_tot_) were found to be in the range from 0.227 to 0.233 cm^3^/g. These materials can be classified as mesoporous, with an average pore diameter (W) of approximately 55 Å (5.35–6.10 nm). The SEM and EDS analyses indicated that the EGDMA/VA microspheres are globular in shape with well-defined edges and contain 73.06% of carbon and 26.94% of oxygen. The microspheres containing modified starch exhibited a loss of smoothness with more irregular shape. The adsorption efficiency of dyes and heavy metal ions depends on the phases contact time, initial adsorbate concentration and the presence of competing electrolytes and surfactants. The equilibrium data were better fitted by the Freundlich isotherm model than by the Langmuir, Temkin, and Dubinin-Radushkevich models. The highest experimental adsorption capacities were observed for the BB3 dye which were equal to 193 mg/g, 190 mg/g, and 194 mg/g for EGDMA/VA, EGDMA/VA-St/B, EGDMA/VA-St/DiTDTA, respectively. The dyes and heavy metal ions were removed very rapidly and the time required to reach system equilibrium was below 20 min for M(II), 40 min for BB3, and 120 min for AG16. 50% v/v methanol and its mixture with 1 M HCl and NaCl for dyes and 1 M HCl for M(II) desorbed these impurities efficiently.

## 1 Introduction

**FIGURE 1:**
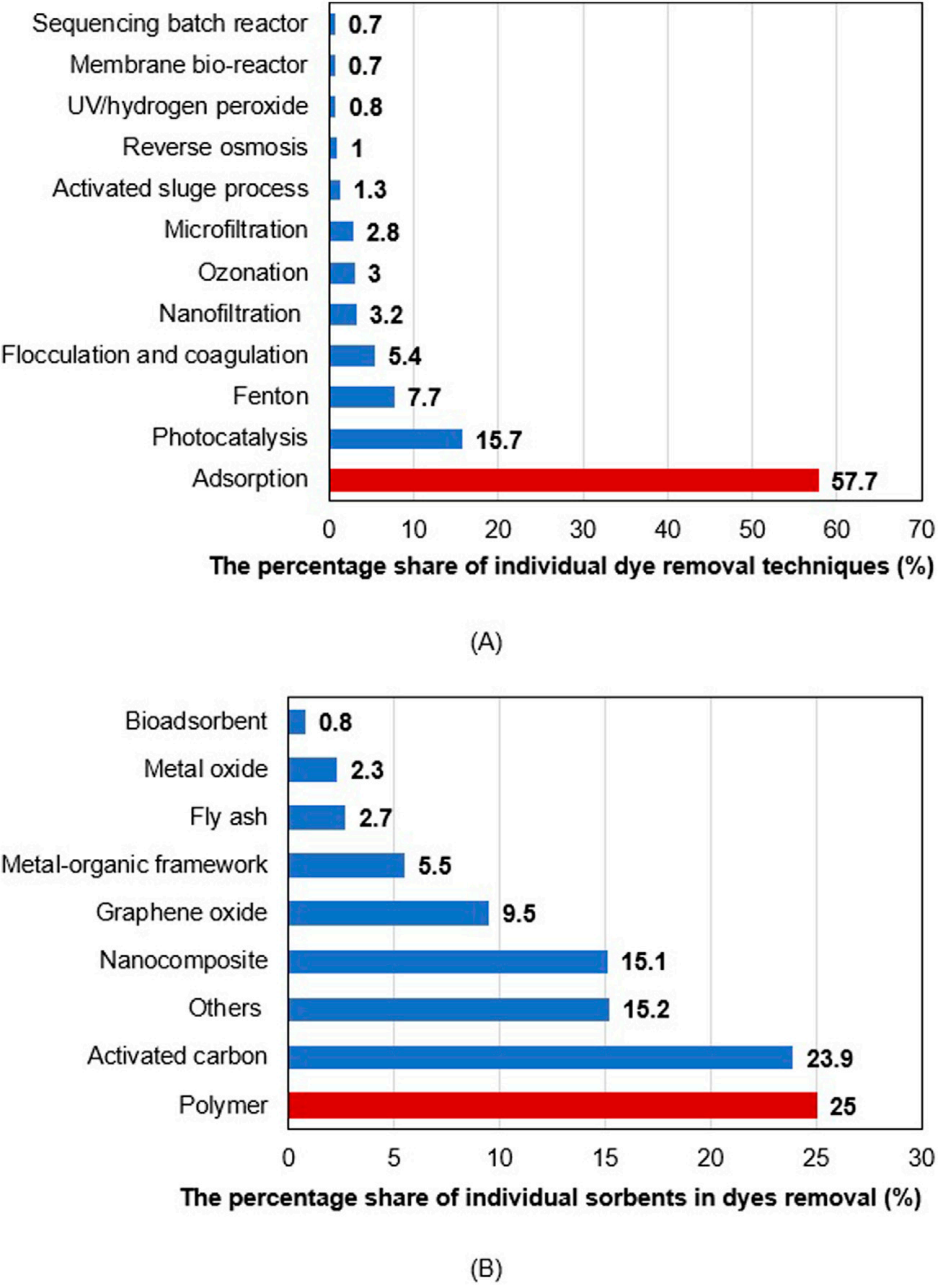
Percentage of literature availiable dyes removal by application of various removal technique **(A)** and sorbents **(B)**.

Although 70% of the Earth’s surface is covered by water, only 2.5% is used as freshwater (Rao et al., 2021). Rapid industrialization, urbanization, and population growth are responsible for huge water consumption and significant water pollution, which has increased the need for clean water (Azeroual et al., 2024; Dutta et al., 2021; Velusamy et al., 2021). Currently, drinking water shortages affect one-third of the world’s population, and it is estimated that by 2025, 1.8 billion people will be affected by water shortages, while according to the World Health Organization, water stress will reach up to 6.0 billion people worldwide (Baghbanzadeh et al., 2017; Boretti and Rosa, 2019). Furthermore, the effects of water scarcity will be exacerbated by global warming and droughts (Baghbanzadeh et al., 2017). In addition, the development and modernization of industry leads to an increase in water pollution as a result of the generation of large amounts of wastewater containing organic and inorganic pollutants such as heavy metals, dyes, and others (Santander et al., 2020; Bensalah et al., 2023). In addition, the development and modernization of industry leads to an increase in water pollution as a result of the generation of large amounts of wastewater containing organic and inorganic pollutants (Santander et al., 2020; Es-Sahbany et al., 2021). In general, industrial activities are the main sources of water pollution (Haq et al., 2022). Among the various organic and inorganic pollutants encountered in wastewater, both dyes and heavy metals are the major pollutants of the aquatic environment of special concern (Varghese et al., 2018; Mishra et al., 2019; Ouass et al., 2024). Wastewater containing dyes and heavy metals cause potential hazards both to the environment and human health (Kahlon et al., 2018; Khan et al., 2023; Mishara et al., 2019; Negahdari et al., 2021; Peng et al., 2022). The main sources of heavy metals and dyes in the environment are industrial wastewater or/and agriculture discharged directly into the water (Khan et al., 2023). Pollutants such as heavy metals mainly come from the metal plating industry, mining, battery manufacturing, or pesticide application whereas the dyes contaminated by textile, tannery, dyeing, paper, and pulp industries (Azeroual et al., 2024; Dutta et al., 2021; Khan et al., 2023; Velusamy et al., 2021). Textile effluents could contain also traces of heavy metals. They can occur naturally in the fibre structure, be introduced into the textile fabric during manufacturing processes, or be components of reactive metal-containing dyes, metal complex acid dyes, chrome acid dyes, and direct metal-containing dyes. Additionally, heavy metals can also come from chemical treatment of fibers (to improve resistance to light and wet) and fabric bleaching processes (as catalysts for oxidants) (Velusamy et al., 2021). The textile industry uses a huge amount of water for fiber processing (steps: fiber desizing, bleaching, mercerizing, fabric dyeing, printing, finishing) which results in the production of huge amounts of effluents and their release into aquatic systems that contain not only dyes, heavy metals but also toxic substances such as inorganic salts, acids, bases, mordants, fastners, fixers, surfactants, defoamers, etc. which further pollute the water systems (Khandare and Govindwar, 2015; Mishra and Maiti, 2020). For example, 1.6 million liters of water are used daily to produce 8,000 kg of fabrics, of which 16% is used in dyeing and about 8% in printing (Khandare and Govindwar, 2015; Velusamy et al., 2021; Jebli et al., 2023). It has been suggested that textile production is responsible for about 20% of global water pollution and nearly 2%–20% of dyes are leached with wastewater (Haq et al., 2022). During dyeing processes, a significant amount of dyes, about ∼15%, remain unfixed with fibers, which accounts for about 280 kilotons of dyes realized in wastewater annually, including 1%–5% lost during production and 1%–10% lost during use (Siddiqui et al., 2019; Mishra et al., 2021). Taking into account industry wastewater the textile industry releases the highest amount of effluents containing dyes (54%) (the dyeing industry - 21%, the pulp and paper industry - 10%, the leather and paint industry - 8%, the dye industry - 7%) (Roa et al., 2021; Velusamy et al., 2021). Both dyes and heavy metal ions can cause serious health problems and impose on the environment and aquatic biota (Kahlon et al., 2018; Khan et al., 2023; Mishara et al., 2019; Negahdari et al., 2021; Peng et al., 2022). For example, synthetic dyes are resistant to biodegradation, light, heat, and oxidizing agents, they can act as mutagenic and carcinogenic agents, cause allergies and skin irritation, liver problems and/or disruption of the human central nervous system, etc. (Haq et al., 2019; Haq et al., 2022). In addition, the presence of dyes in water results in light absorption, reduced photosynthetic activity of algae and aquatic plants, and interruption of the food chain (Haq et al., 2022; Al-Tohamy et al., 2022). As reported by the Ecological and Toxicological Association of the Dyestuffs Manufacturing Industry based on a survey study on the ecotoxicity of textile dyes, ≥90% of the dyes used in the fabric dyeing process have a lethal dose value, *LD*_*50*_ ≥ 2000 mg/kg (Mishra et al., 2021; Vikrant et al., 2018). The AG16 dye discussed in this paper is a dye of the triphenylmethanes class used to dye silk, wool, nylon, and cotton, as well as leather and paper production. It is considered to be potentially toxic to humans and animals. The presence of AG16 in water causes both sunlight absorption and interfering biological processes (Foguel et al., 2017; Rossatto et al., 2020). Moreover, toxicology studies have already been shown its mutagenic and genotoxic effects in mice (Foguel et al., 2017; Wrońska-Nofer et al., 1997). The BB3 cationic dye applied in the textile industry (also discussed in this paper) is used to dye cotton, silk, wool, and other fibers. It is characterized by excellent light-fastness ensuring resistance to color fade (Fobiri, 2022). It could be very toxic to aquatic life, and cause serious eye irritation, and damage (Al-Tohamy et al., 2022). The heavy metals introduced into the aquatic environment from anthropogenic sources accumulate in fish gills, and algae, enter the food chain, and affect human life causing various diseases including damage to organs such as kidneys and liver, Alzheimer’s disease, disturbance in the nervous system, anemia, etc. (Haq et al., 2022; Mitra et al., 2022). Due to the widespread use of dyes and heavy metals, their properties, and their impact on the environment, their removal from wastewater is of utmost importance for the protection of health and the environment (Qasem et al., 2021). Research in this area is ongoing, and the importance and relevance of this topic is evidenced by the increase in the number of published scientific articles on “dyes removal” (Dutta et al., 2021) and “heavy metals removal” (Burakov et al., 2018; Nayeri and Mousavi, 2024). Based on a literature review of dye removal by different removal techniques (search by keywords: dye, treatment, water, name of removal technique), it was found that adsorption (Figure 1A) and among the sorbents (search by keywords: dye, adsorption, water, type of adsorbent used) polymers play a significant role (Figure 1B) (Dutta et al., 2021).

The adsorption method is the most widely used and favorable among the other removal methods due to its simple procedure, low time consumption, widespread availability of various types of adsorbents, high efficiency and/or selectivity, relatively low cost, and sludge-free process. In addition, the ability to recycle and reuse adsorbent material is another advantage of adsorption (Haq et al., 2022; Dutta et al., 2021). On the other hand, due to polymer properties such as easy fabrication, multifunctionality, high effective surface area, high surface-to-volume ratio, large number of active sites, reusability, low cost, high reactivity and high efficiency towards dyes and heavy metals make these adsorbents very popular (Dutta et al., 2021). In the literature, different adsorbents are tested for the removal of dyes (Dutta et al., 2021; Yagub et al., 2014; Zhou et al., 2019) and heavy metal ions (Chakraborty et al., 2020; Dutta et al., 2021; Qasem et al., 2021) removal but some of them are expensive and not renewable. Therefore biopolymers as widely available, renewable, and cost-effective materials have played a more significant role in polymer substitution nowadays (Dutta et al., 2021; Doughmi et al., 2024). Starch is one of the most abundant, economically viable, renewable, biodegradable, non-toxic in nature, naturally occurring polysaccharides. Due to its properties has been extensively used in different fields among others in the removal of dyes and heavy metal ions from water and wastewater (Gupta et al., 2021; Haq et al., 2022; Ihsanullah et al., 2022; Majeea et al., 2024; Wawrzkiewicz et al., 2025). Starch is a homopolysaccharide of glucose consisting of amylose and amylopectin. The amylose and amylopectin account for 15%–30% and 70%–85%, respectively, of native starch (usually in a 1:3 ratio) (Gupta et al., 2021; Ihsanullah et al., 2022; Majeea et al., 2024). Amylose is a linear polymer in which D-glucose molecules are linked by α-(1-4)-glycosidic linkages, while amylopectin has additional α-(1-6)-glycosidic linkages that cause its polysaccharide backbone to be branched (Haq et al., 2019; Majeea et al., 2024). However, as reported, the applications of native starch are limited due to low solubility in organic solvents, loss of viscosity, low fluidity, tendency to retrogradation, low thermal stability, gelatinization temperature, low paste clarity, and high gel turbidity (Kumari and Sit, 2023). Replacement of active hydroxyl groups of native starch with other functional groups during modification processes allows us to overcome these drawbacks and shortcomings. Modification of native starch is indispensable to meet industrial requirements, which results in increased dyes and heavy metal adsorption ability and extended application. Modification of starch offers numerous benefits including improved functionality and textural properties, improved stability, and increased versatility (Kumari and Sit, 2023; Dabagh et al., 2023). The modification of starch has usually been carried out in physical, chemical, genetic, and enzymatic ways to obtain better properties in all aspects compared to the native starch (Haq et al., 2019; Haq et al., 2022; Kumari and Sit, 2023). Taking into account the abovementioned properties, the starch for new adsorbents that meet strictly defined criteria for removing dyes and heavy metal ions to protect health and the environment is still a great challenge. To meet these challenges the aim of this work was to synthesize new polymer microspheres based on ethylene glycol dimethacrylate (EGDMA) and vinyl acetate (VA) (EGDMA/VA) without and with starch (St) as biocomponent modified with boric acid (B) or dodecyl-S-thiuronium dodecylthioacetate (DiTDTA), designated as EGDMA/VA, EGDMA/VA-St/B and EGDMA/VA-St/DiTDTA, respectively. First, the synthesized adsorbents were characterized by the attenuated total reflectance fourier transformed infrared spectroscopy (ATR/FT-IR), scanning electron microscope (SEM), differential scanning calorimetry (DSC), porous structure, and point of zero charge (pH_PZC_). Then their removal of C.I. Basic Blue 3 (BB3) and C.I. Acid Green 16 (AG16) dyes and heavy metal ions M(II) such as Cu(II), Ni(II), Zn(II) from waters and wastewaters ability were tested. The effect of initial adsorbate concentration, phases contact time, pH, competing electrolytes, and surfactants on dyes and heavy metal ions removal efficiency were taken into account. The kinetic, isotherm, and desorption studies were presented and the equilibrium data were fitted using the Langmuir, Freundlich, Temkin, and Dubinin-Radushkevich isotherm. This research’s innovative and interdisciplinary character should be emphasized, as it originates from the borderline between chemical technology, materials engineering, and environmental protection. The issues discussed here in the field of preparation, physicochemical characteristics, and application of the new polymer microspheres in the environmental aspect seems to be extremely important from a cognitive, technological, and ecological point of view and can ultimately contribute to a significant improvement of the state of knowledge in the analyzed field and to the development of technological assumptions for the implementation of this process. Reducing the toxicity of the discharged effluent or increasing the spectrum of handling options can also be a measurable effect of this research.

## 2 Experimental

### 2.1 Chemicals

The following reagents and materials were used in this study:

Chemicals for polymer microspheres preparation: soluble starch, calcium chloride (CaCl_2_), sodium hydroxide (NaOH), (Chempur, Poland); benzyl alcohol, acetone, (Avantor Performance Materials Poland S.A., Poland); ethylene glycol dimethacrylate (EGDMA), vinyl acetate (VA), poly(vinyl alcohol) (PVA), α,α′-azobis(isobutyronitrile) (AIBN), boric acid (B), thiourea, dodecyl-S-isothiuronium bromide, dodecylthioacetic acid (Sigma-Aldrich Germany); dyes: C.I. Basic Blue 3 (BB3), C.I. Acid Green 16 (AG16) (Sigma-Aldrich company, Germany); heavy metals: copper(II) chloride dihydrate salt (CuCl_2_·2H_2_O), nickel(II) chloride dihydrate salt (NiCl_2_·2H_2_O), zinc(II) chloride salt (ZnCl_2_) (Chempur, Piekary Sląskie, Poland); desorption agents: hydrochloric acid (HCl), nitric(V) acid (HNO_3_), sulfuric(VI) acid (H_2_SO_4_), sodium hydroxide (NaOH), ammonia aqueous solution (NH_3_·H_2_O) (Chempur, Piekary Sląskie, Poland); sodium chloride (NaCl), methanol (MeOH) (Avantor Performance Materials Poland S.A., Poland); auxiliary substances: anionic surfactant sodium dodecyl sulfate (SDS), non-ionic surfactant t-octylphenoxypolyethoxyethanol (Triton X100, TX100) (Sigma-Aldrich company, Germany); sodium chloride (NaCl), sodium sulphate (Na_2_SO_4_) (Avantor Performance Materials Poland S.A., Poland).

**FIGURE 2:**
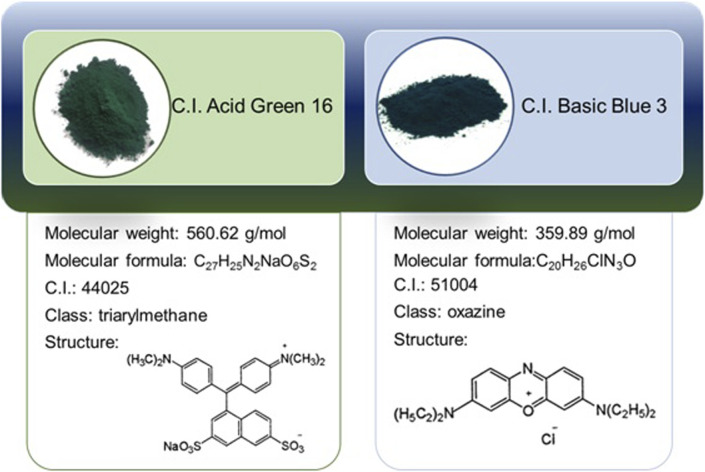
Background information on applied dyes.

BB3 and AG16 stock solutions were prepared by dissolving the appropriate amounts of the dyes in distilled water in a volumetric flask. Working solutions of BB3 and AG16 were made by diluting the stock solutions. Dyes characteristic is presented in Figure 2.

The reagents used in the study had analytical grade purity.

Heavy metals stock solutions were prepared by dissolving appropriate amounts of heavy metal salts in 0.1 M HCl. Working solutions of appropriate concentrations were prepared from the stock solutions by dilution to obtain the desired concentrations of Cu(II), Ni(II), Zn(II). In addition, HCl or NaOH solutions of 1 M concentration were used to adjust the pH of the appropriate solutions. The concentration of dyes and heavy metal ions was selected to reflect their actual concentrations in real wastewater samples.

### 2.2 Preparation of EGDMA/VA, EGDMA/VA-St/B and EGDMA/VA-St/DiTDTA beads

#### 2.2.1 Synthesis of dodecyl-S-isothiuronium dodecylthioacetate (DiTDTA)

Dodecyl-S-isothiuronium dodecylthioacetate was obtained by reacting dodecyl-S-isothiuronium bromide with dodecylthioacetic acid in sodium hydroxide. Dodecylthioacetic acid was obtained in an earlier step from dodecyl bromide and thiourea, and then from the resulting dodecyl-S-isothiuronium bromide and chloroacetic acid in the presence of an aqueous solution of sodium hydroxide.

#### 2.2.2 Modification of starch by boric acid (St/B)

Soluble starch was modified using a mechanochemical method. 15.00 g of starch was mixed and highly grated in a mash pot with 6.20 g (0.10 mol) of boric acid for 45 min. The grated mixture was then dried-heated in a chamber at 95–98°C for 10 h, to constant weight. Cyclically, the grinding - mixing of the ingredients was repeated every 2 hours.

#### 2.2.3 Modification of starch by dodecyl-S-isothiuronium dodecylthioacetate (St/DiTDTA)

Soluble starch (St) modification was carried out using a mechanical method. 1.00 g of soluble starch was mixed and highly grated in a mash pot with 2.00 g dodecyl-S-isothiuronium dodecylthioacetate for 45 min. The grated mixture was then dried-heated in a chamber at 60–65°C for 5 h, to constant weight.

#### 2.2.4 Preparation of polymeric microspheres with starch (St)

**FIGURE 3:**
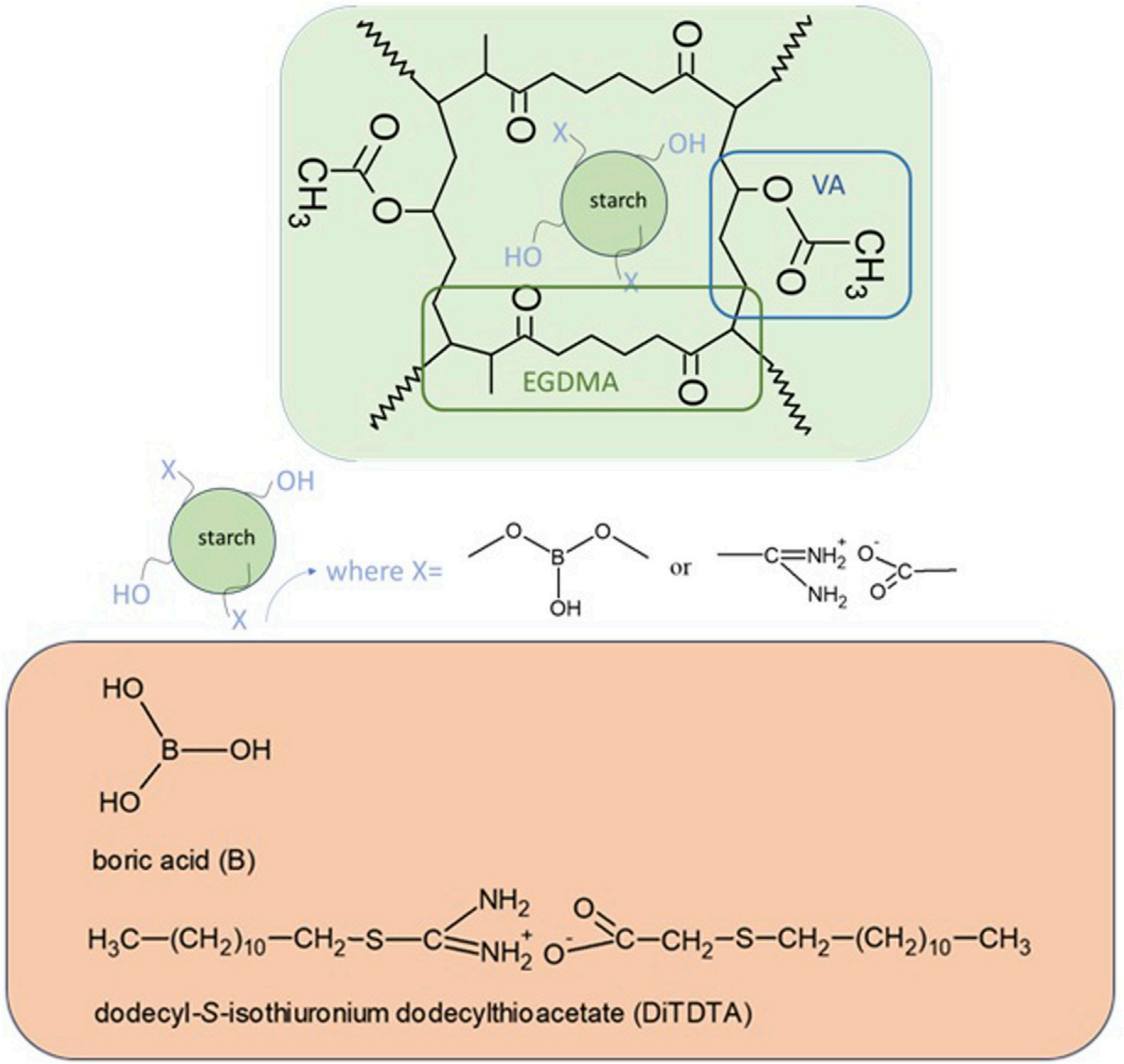
Chemical structures of monomers and modified starch used to synthesize microspheres.

Polymeric microspheres were synthesized using the suspension-polymerization methodology (Gawdzik et al., 2006; Podkościelna et al., 2006; Podkościelna and Gawdzik, 2010; Wawrzkiewicz et al., 2020). 1.00 g of poly(vinyl alcohol) was dissolved in 150 mL of redistilled water. Next, 1.20 g CaCl_2_ was added and the whole content was stirred for 1.5 h at 80°C in a three-necked flask fitted with a thermometer, a water condenser, and a mechanical stirrer. 3.00 g of starch of different types (Table 1) was previously dissolved in 15 mL of benzyl alcohol (2 h at 60°C). Then, the organic mixture containing: 10.00 g of the EGDMA and 4.30 g of VA (in molar mass 1:1), 1% wt. of initiator AIBN, and the starch with benzyl alcohol was added to the intensively stirred aqueous medium. Copolymerization was continuous for 12 h at 80–85°C. The obtained microspheres were washed with distilled water (2000 mL). The chemical structures of monomers and the proposed fragment of copolymer structure with modified starch are presented in Figure 3. Microspheres were dried at 80°C to a constant weight.

**TABLE 1:** Experimental parameters of polymeric adsorbents synthesis.

Sample No	EDGMA (g)	VA (g)	Starch (g) (St/B)	Starch (g) (St/DiTDTA)	AIBN (g)
1	10.00	4.30	—	—	0.15
2	10.00	4.30	3.00	—	0.15
3	10.00	4.30	—	3.00	0.15

Where: St/B–soluble starch modified with boric acid; St/DiTDTA–soluble starch modified with dodecyl-*S*-isothiuronium dodecylthioacetate.

### 2.3 Evaluation of the physicochemical properties of EGDMA/VA, EGDMA/VA-St/B and EGDMA/VA-St/DiTDTA

The Fourier transform infrared spectra were recorded with a Bruker Tensor 27 FTIR spectrometer (Germany) using the attenuated total reflectance technique. The samples were thin films. All spectra were obtained at room temperature after averaging 32 scans between 600 and 4,000 cm^−1^ with a resolution of 4 cm^−1^ in the absorbance mode.

Differential scanning calorimetry measurements were performed with a Netzsch DSC 204 calorimeter (Germany) operated in the dynamic mode. DSC measurements were made using aluminum pans with pierced lids and a sample mass of ∼5–10 mg under a nitrogen atmosphere (30 mL/min). Dynamic scans were performed at a heating rate of 10 C/min in the temperature range of −20°C–600°C. The parameters such as the range of decomposition temperatures (T_onset,_ T_offset_) and maximum decomposition temperature (T_d_) were determined.

The porous structures of the copolymers were characterized by N_2_ adsorption at −196°C (ASAP 2405 adsorption analyzer, Micrometrics Inc., United States). Before the analysis, the copolymers were degassed at 120°C for 2 h. The specific surface area was calculated according to the Brunauer-Emmett-Teller (BET) method, assuming that the area occupied by a single nitrogen molecule is 16.2 Å^2^. The pore volumes and pore size distributions were determined by the Barrett-Joyner-Halenda (BJH) method.

The scanning electron microscope Quanta 3D FEG with the acceleration voltage 20 kV coupled with energy dispersive X-ray spectroscopy (EDS) (FEI, US) was applied for visualization of morphology and microstructure of starch-modified adsorbents as well as their composition.

The pH_PZC_ (where PZC is the point of zero charge) value of EGDMA/VA, EGDMA/VA-St/B, and EGDMA/VA-St/DiTDTA adsorbents was determined by the drift method (Kosmulski, 2023). 50 mL of 0.01 M KNO_3_ solution was equilibrated for 24 h with 0.05 g of polymeric adsorbents in a laboratory shaker Elpin +358S (Elpin, Lubawa, Poland). The initial pH (pH_0_) of the solution was adjusted from 2 to 10 using 1 M NaOH or 1 M HCl. Then, the final pH (pH_f_) was measured using a pH-meter CPC-411 (Elmetron, Poland). Plotting ΔpH (ΔpH = pH_0_ – pH_f_) *versus* pH_0_ it is possible to obtain pH_PZC_ of polymeric adsorbent.

### 2.4 Adsorption and desorption experiments

Adsorption studies were carried out using the batch method at room temperature. The impact of the different parameters, influencing the adsorption phenomenon, was studied in the following ranges: dye concentrations (1–500 mg/L), metal ion concentrations (5–120 mg/L), pH (5–7), electrolytes (NaCl, Na_2_SO_4_) concentration (5 g/L), surfactants (SDS, TX100) concentration (0.5 g/L), time (1–240 min (kinetic tests) or 24 h (equilibrium tests)). The pH of the solution was adjusted using 1 M NaOH and 1 M HCl. In each experimental step, 0.05 g of the adsorbents were placed in 20 mL of dyes or metal ions aqueous solutions. The flasks were positioned in an Elpin +358S mechanical shaker (Elpin, Poland) with a constant oscillation amplitude (A = 8) and rotation of 150 cycles/min selected on the basis of a previously conducted optimization process. Adsorption and desorption experiments were carried out in triplicate and the mean value of the results (reproducibility ±5%) was used to evaluate the data.

The amounts of dyes or metal ions sorbed by EGDMA/VA, EGDMA/VA-St/B, and EGDMA/VA-St/DiTDTA at specific time t (q_t_) and at equilibrium (q_e_), also known as adsorption capacity was calculated from Equations 1, 2:

$$q_{t}=\frac{\left(C_{0}-C_{t}\right)V}{m}$$ (1)

$$q_{e}=\frac{\left(C_{0}-C_{e}\right)V}{m}$$ (2)

where C_0_, C_t,_ and C_e_ (mg/L) are the concentrations of dyes or metal ions before adsorption, after specific time intervals, and at equilibrium, respectively; V (L) is the volume of solutions; m (g) is the weight of the EGDMA/VA, EGDMA/VA-St/B and EGDMA/VA-St/DiTDTA.

After the separation of the solution from the adsorbents by the filtration, the determination of the content of dyes and metal ions was carried out by ultraviolet-visible (UV–vis) spectrophotometry (Carry 60, United States) or atomic absorption spectroscopy (AAS) (Varian AA240FS, Australia) methods. Parameters of the UV-vis spectrophotometer were as follows: 1 cm quartz cell, maximum absorbance wavelength of 639 nm for AG16 and 654 nm for BB3, split width 1 mm, and integration time 1 s. Using the fast sequential atomic absorption spectrometer with SIPS autosampler the concentrations of heavy metal ions were measured at wavelengths of 232.0 nm, 324.8 nm, and 213.9 nm; the lamp currents were 4 mA; 4 mA, 5 mA; slit widths were 0.2 nm, 0.5 nm, 1 nm; air/acetylene flow was 13.5/2 L/min for Cu(II), Ni(II), and Zn(II), respectively.

The following methodology was employed in the regeneration experiments: the adsorbed EGDMA/VA, EGDMA/VA-St/B, and EGDMA/VA-St/DiTDTA beads (0.05 g) with dyes or heavy metal ions were shaken (A = 8, and 150 cycles/min.) for 3 h with different elution solutions (20 mL) to remove the adsorbed dyes and metal heavy metal ions. The composition of eluting solutions for dyes removal from the polymer phase was: 1 M HCl, 1 M NaOH, 1 M NaCl, 50% v/v MeOH, 1 M HCl+50% v/v MeOH, 1 M NaOH+50% v/v MeOH, 1 M NaCl+50% v/v MeOH. Metal ions were eluted from the adsorbent phase using such solutions as 1 M HCl, 1 M HNO_3_, 1 M H_2_SO_4_, 1 M NH_3_·H_2_O and 1 M NaOH. The content of desorbed dyes and metal ions in the solution was then determined and the percentage of desorption (D) was calculated from Equation 3:

$$D=\frac{m_{des}}{m_{ads}}100\%$$ (3)

where m_des_ (mg) is the mass of desorbed dyes or metal ions; m_ads_ (mg) is the mass of sorbed dyes or metal ions.

## 3 Results and discussion

### 3.1 Polymers characterization

#### 3.1.1 Attenuated Total Reflectance Fourier Transformed Infrared Spectroscopy (ATR/FT-IR)

**FIGURE 4:**
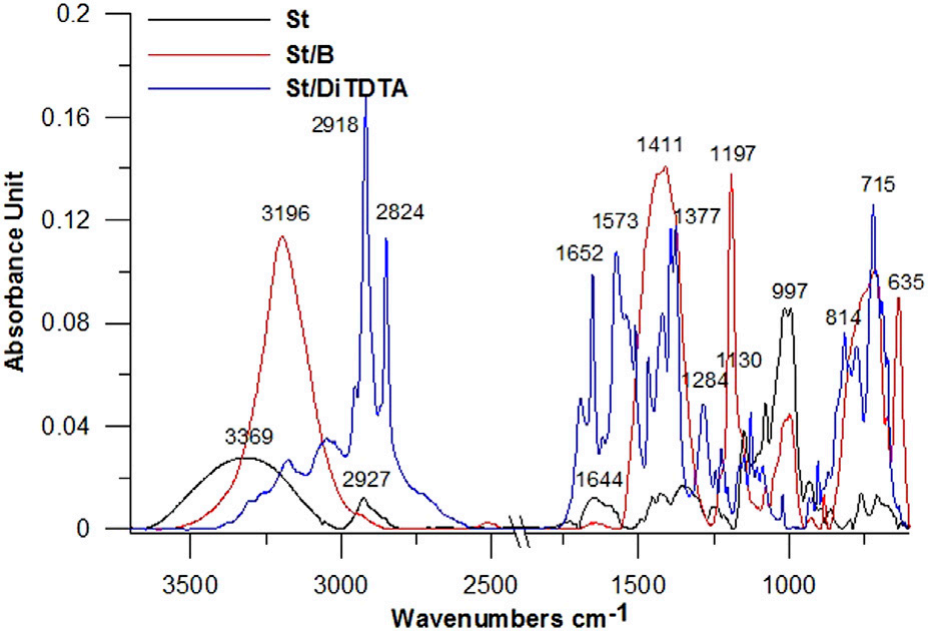
ATR-FTIR spectra of starch before and after modifications.

The first stage of work include the modification of the original starch with boric acid and dodecyl-*S*-isothiuronium dodecylthioacetate. To confirm the correct course of the modification the ATR/FT-IR spectra were performed (Figure 4).

On the original starch spectra, the wide absorption band around 3,015–3,550 cm^−1^ is assigned to the stretching vibrations of hydroxyl groups (O-H). The signal around 2,927 cm^−1^ is attributed to the stretching vibrations of the C-H aliphatic (Kizil et al., 2002; Pozo et al., 2018). The signals at 1,430 and 1,350 cm^−1^ correspond to -CH of a saturated system. In turn, the signals 1,152 and 1,003 cm^−1^ are assigned to C-O groups. The 920, 770, and 722 cm^−1^ vibrations are assigned to the pyranose system. Additionally, the signal at 1,644 cm^−1^ can be assigned to water adsorbent in the amorphous region of starch (Fang et al., 2002; Nzenguet et al., 2018).

In the case of starch modification with boric acid, the regions 3,500–2,950 and 3,196 cm^−1^ correspond to C-H, -CH_2,_ and -CH groups. The signal at 1,411 cm^−1^ can be assigned to C-CH, OH, -O-B = groups. The strong signals: 997, 884, and 635 cm^−1^ indicate the interaction of starch with boric acid (Tiwari et al., 2013). The vibrations at 920, 770, and 715 cm^−1^ are assigned to the pyranose system.

In the spectra of dodecyl-S-isothiuronium dodecylthioacetate derivative, the absorption at 3,304 cm^−1^ and 3,177 cm^−1^ is due to N-H and C-H symmetric and anti-symmetric stretching vibration. The peak at 2,954, 2,918, and 2,850 cm^−1^ represent the -CH, -CH_2_ groups. The characteristic signal 1,693 cm^−1^ is assigned to C=O (COO-). The signal at 1,652 cm^−1^ represents the C=N bending vibrations. The absorption peaks at 1,468 cm^−1^ and 1,105 cm^−1^ are due to N-C-N anti-symmetric and symmetric stretching vibration. The peaks at 1,244 and 720 cm^−1^ are due to C-S-C stretching and C-S symmetric stretching, respectively (Hemalatha et al., 2006).

**FIGURE 5:**
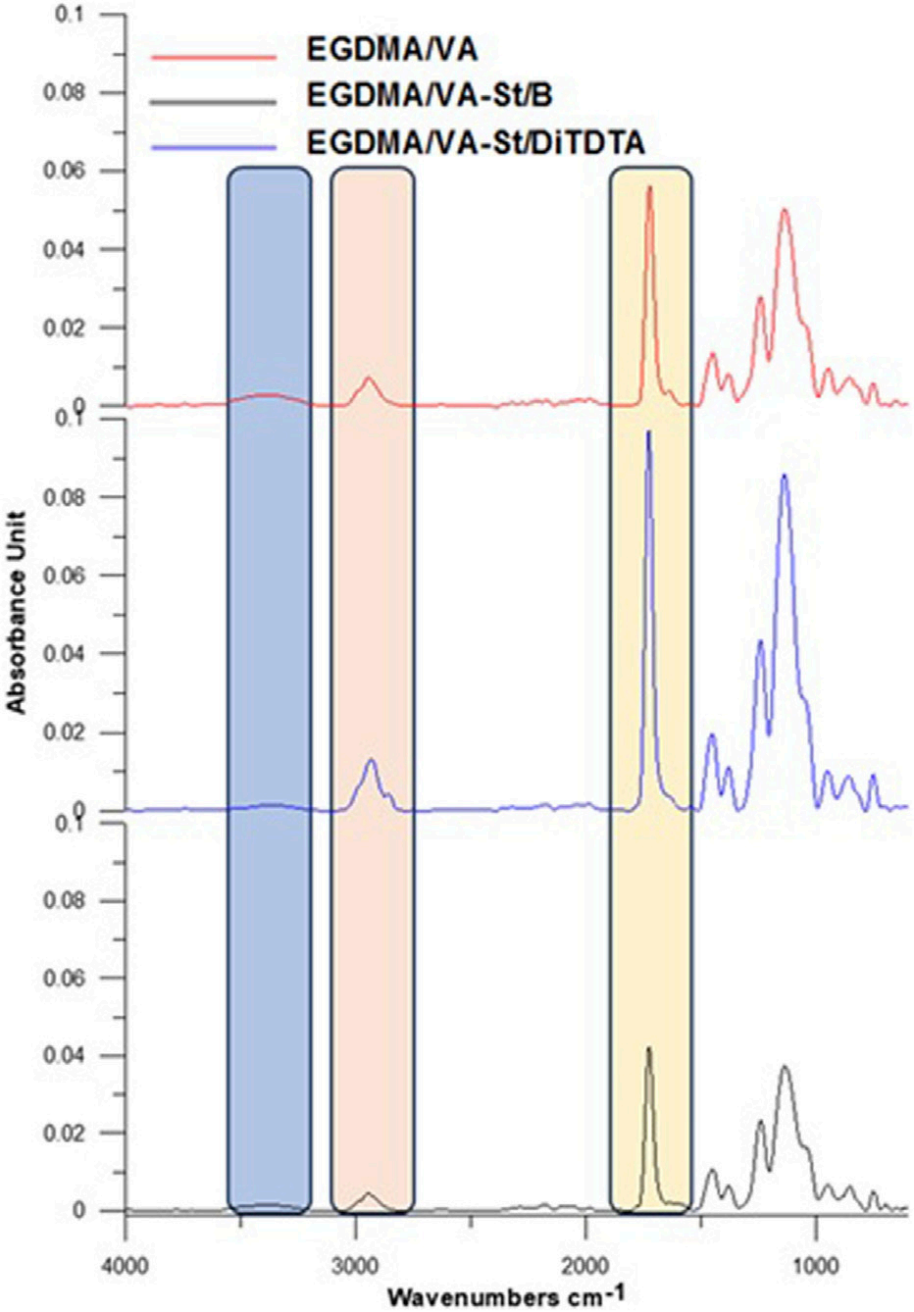
ATR-FTIR spectra of polymeric microspheres.

To confirm the structure of polymeric microspheres, ATR/FT-IR spectra were performed for all copolymers (Figure 5). The most important signals have been marked on the curves, their course being closely related to the structure of the monomers: EGDMA and VA. In the spectra in the range at 3,450 to 3,300 cm^−1^ the O-H hydroxyl groups are observed. The intensity of the signal decreases after the modification of starch. The signal around 2,945–2,931 cm^−1^ is qualified to stretching vibrations in C-H aliphatic. The characteristic sharp signal around 1720 cm^−1^ is assigned to C=O stretching vibrations. The signals -C-OH and C-O stretching vibration groups (about 1,130, 1,050 cm^−1^) are observed.

#### 3.1.2 Differential scanning calorimetry (DSC)

**FIGURE 6:**
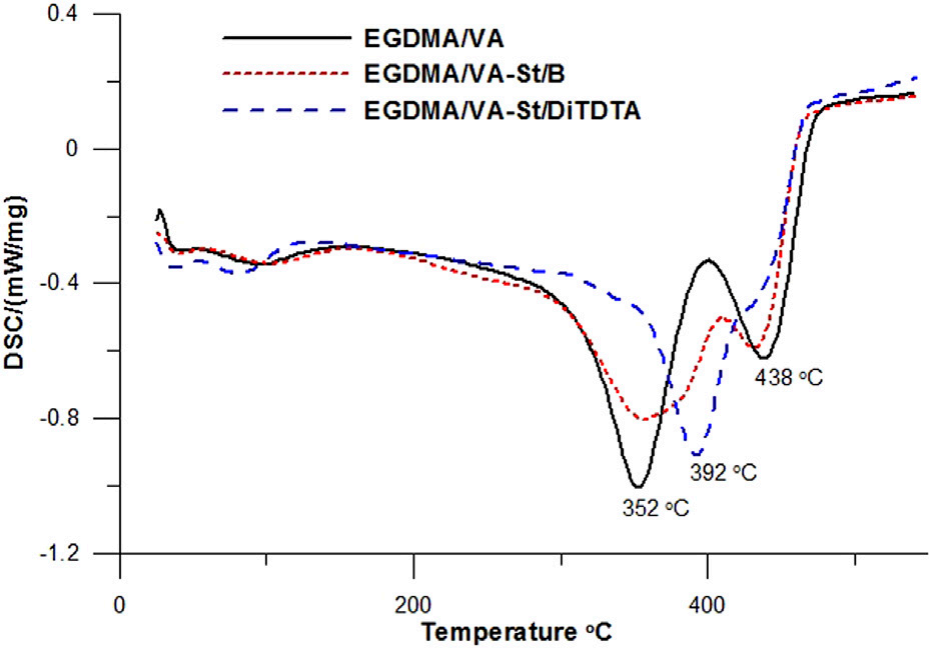
DSC curves of the obtained polymeric microspheres.

To evaluate the thermal resistance of the microspheres the DSC analysis was done. The DSC curves for microspheres with modified starch and parent EGDMA/VA copolymers are presented in Figure 6. On the curves, two endothermic effects (352 and 432–438°C) associated with the polymeric microspheres’ thermal degradation process (T_d_) are visible. The first endothermic effect corresponds to the degradation of linear ester groups. The second endothermic effect is involved in the degradation of crosslinked polymeric fragments. Only the derivative with the addition of DiTDTA has a different course. In the DSC curve, we can see one clear endothermic signal (392°C) associated with the total sample degradation. The addition of modified starch increases the thermal resistance of microspheres.

#### 3.1.3 Scanning electron microscopy (SEM) and energy dispersive X-ray spectroscopy (EDS)

**FIGURE 7:**
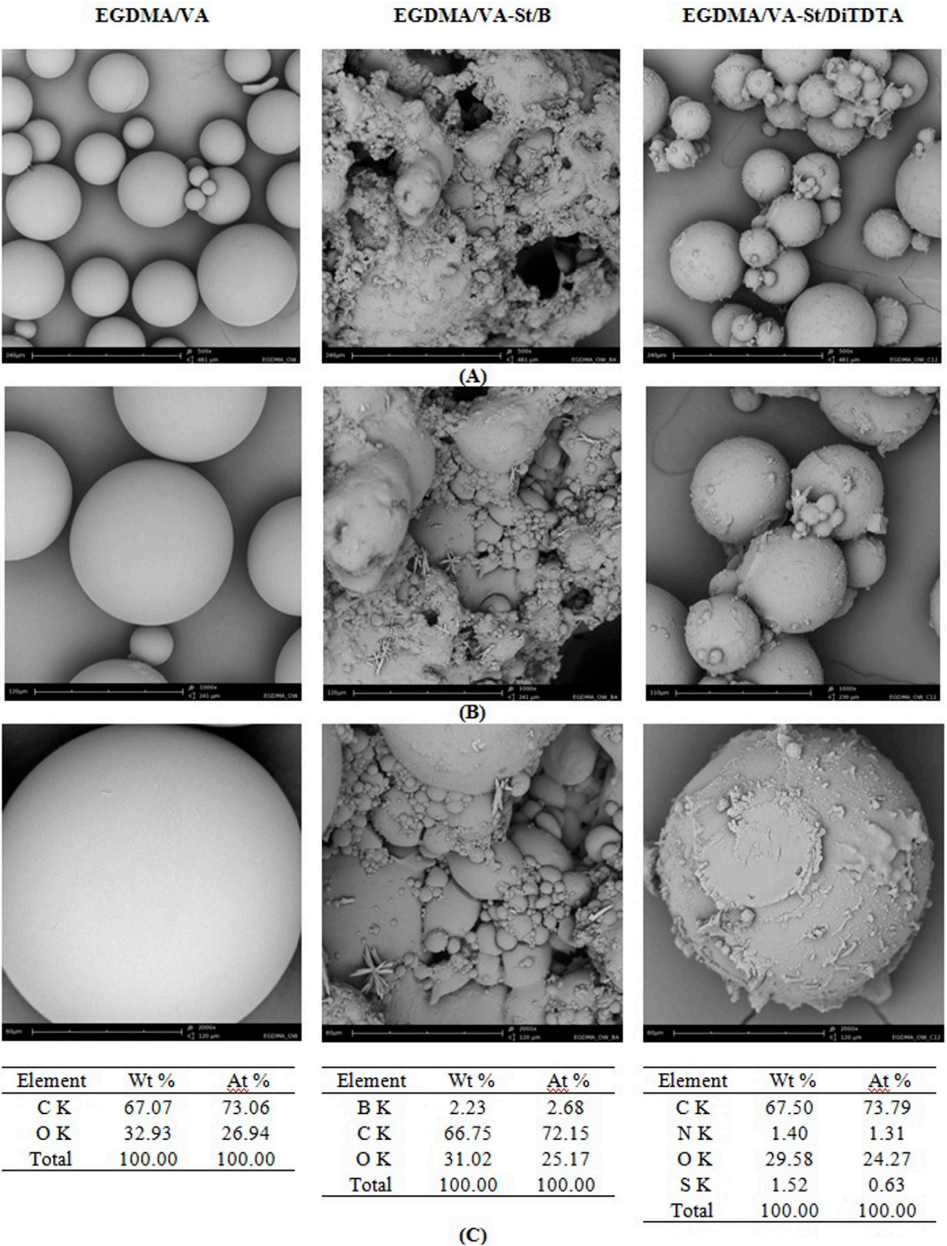
SEM micrographs of EGDMA/VA, EGDMA/VA-St/B and EGDMA/VA-St/DiTDTA adsorbents at varying magnifications: **(A)** ×500, **(B)** ×1,000, and **(C)** ×2000.

The scanning electron microscopy and energy-dispersive X-ray spectroscopy analysis elucidated the morphological characteristics and the chemical composition of the EGDMA/VA, EGDMA/VA-St/B, and EGDMA/VA-St/DiTDTA adsorbents. Figure 7 depicts the SEM micrographs of the EGDMA/VA, EGDMA/VA-St/B, and EGDMA/VA-St/DiTDTA adsorbents at varying magnifications. The SEM analysis of the EGDMA/VA microspheres revealed that these microspheres are globular in shape with well-defined edges. The microspheres are observed to be smooth in shape and to possess varying sizes. EDS analysis indicated the presence of 73.06% of carbon and 26.94% of oxygen content in EGDMA/VA. The microspheres containing starch as a biocomponent modified with boric acid and dodecyl-S-thiuronium dodecylthioacetate exhibited a loss of smoothness. The surface of these microspheres is more irregular, wrinkled, rough and discontinuous, especially in the case of the EGDMA/VA-St/B sample. As reported in the literature, boric acid acts as a cross-linking agent for starch, resulting in an improvement of the mechanical and thermal properties of the blend. At a constant starch: PVA ratio, the cross-linking is directly proportional to the boric acid concentration, whereas an increase in the starch: VA ratio at a constant boric acid concentration increases the mechanical and thermal properties of the blend (Gadhave et al., 2018). In addition, analogous to our results, the morphology of adsorption materials, starch films, etc. was changed after modification by boric acid. For example, comparing the morphology of the unmodified thermoplastic starch film with that after boric acid incorporation, changes in the morphology of these materials were observed. The addition of 0.5% and 2% boric acid makes the morphology smoother and more homogeneous compared to unmodified material. The boric acid as a cross-linking agent improves the compatibility and interfacial interactions between starch networks. At 8% boric acid content, the morphology is rougher compared to pristine film (Sun et al., 2018). It can be interpreted as a consequence of damage to starch granules as a result of the reaction (Sun et al., 2018; Yoğurtçu and Gürler, 2024). The increase in surface roughness may also be a result of the oxidation process (boric acid + hydrogen peroxide) (Xie and Wang, 2011). In another study, it was also observed that the mixture of thermoplastic starch/anthocyanin after the addition of boric acid has surface roughness due to the surface interaction between the starch and boric acid (Li et al., 2021a). Wrinkled, rough and discontinuous surface was also observed for the films crosslinked to hydroxypropyl distarch phosphate/polyhydroxyalkanoate composite with boric acid. The EDS confirmed the successful starch modification by the boric acid and the composition of the EGDMA/VA-St/B microspheres was as follows 2.68% of boron, 72.15% of carbon and 25% of oxygen. The EGDMA/VA-St/DiTDTA microspheres are predominantly spherical with a more rough surface and a greater number of indentations compared to the EGDMA/VA microspheres. The formation of these indentations on the surface of the particles may facilitate larger contact areas with heavy metals and dyes than those microspheres with smooth surfaces, which could influence the adsorption capacity of these contaminants. The EDS shows 73.79% of carbon, 1.31% of nitrogen, 24.27% of oxygen and a trace amount of sufur (0.63%).

A well-developed surface area and micro- and mesopores are significant for effective sorption processes. The characteristic parameters of the porous structures of the examined microspheres are presented in Table 2. The largest specific areas are observed for the microspheres found in the parent EGDMA/VA sample (207 m^2^/g). With the addition of modified starch, the decrease in S_BET_ is noticeable (169–184 m^2^/g). This phenomenon relates to the stopping of the microsphere pores by the big starch molecule. These observations are also confirmed by our earlier article (Wawrzkiewicz et al., 2025). These materials can be qualified as mesoporous, the average pore diameter (W) is c.a. 55 Å (5.35–6.10 nm).

**TABLE 2:** Parameters of the porous structures of the studied microspheres.

Adsorbent	Specific surface area, S_BET_ (m^2^/g)	Pore volume, V_TOT_ (cm^3^/g)	Average pore diameter, W (nm)	The most probable pore diameter (nm)
EGDMA/VA	207	0.231	5.35	3.80
EGDMA/VA-St/B	184	0.233	5.07	4.00
EGDMA/VA-St/DiTDTA	169	0.227	6.10	4.50

### 3.2 Adsorption ability of EGDMA/VA, EGDMA/VA-St/B and EGDMA/VA-St/DiTDTA

#### 3.2.1 Effect of pH

It is well known that pH plays a crucial role in the sorption of metal anions and cations because pH influences both the ionization of chemically active sites on the adsorbent and the chemical speciation of metals in aqueous solution. Thus, adsorbent characterization taking into account the protonation/deprotonation behavior of the sorbent material in an aqueous solution could be useful to explain and describe the sorption mechanism (Fiol and Villaescusa, 2008). The determination of pH_PZC_ is also crucial for the dyeing and finishing processes of fibers and for the dye adsorption on fibers (Giacomni et al., 2017). During the dyeing processes, the pH of the dye bath influences, on the one hand, the ionic charge of the fiber in which it is immersed and, on the other hand, the dye adsorption on this fiber, which also depends on the pH_PZC_ of the sorbent. Adsorbents characterized by low pH_PZC_ values are more suitable for the adsorption of cationic dyes, while adsorbents with high pH_PZC_ values are more suitable for the adsorption of anionic dyes. For example, the adsorption of the acid-type dye, used for dyeing silk and wool fibers, on the protein fibers depends on the pH. The samples dyed at pH > pH_PZC_ showed higher color strength compared to the samples dyed at pH < pH_PZC_, which demonstrated higher dye adsorption efficiency (Fiol and Villaescusa, 2008; Giacomni et al., 2017).

The knowledge of the point of zero charge allows us to determine the ionization of adsorbent functional groups and their possible interactions with metal ions or dye species in the studied system. The pH at which the adsorbent surface charge takes a zero value, defined as the point of zero charge, means that the charge of positive and negative surface sites is equal. At solution pH higher than pHpzc adsorbent surface is negatively charged, whereas at pH lower than pHpzc adsorbent surface is positively charged. This means that in the first case (pH > pH_PZC_) the interactions of the adsorbent with positive metal, dye species are more favorable, while in the second case (pH < pH_PZC_) with negative metal, dye species (Fiol and Villaescusa, 2008; Giacomni et al., 2017; Guilhen et al., 2022; Kosmulski, 2023).

**FIGURE 8:**
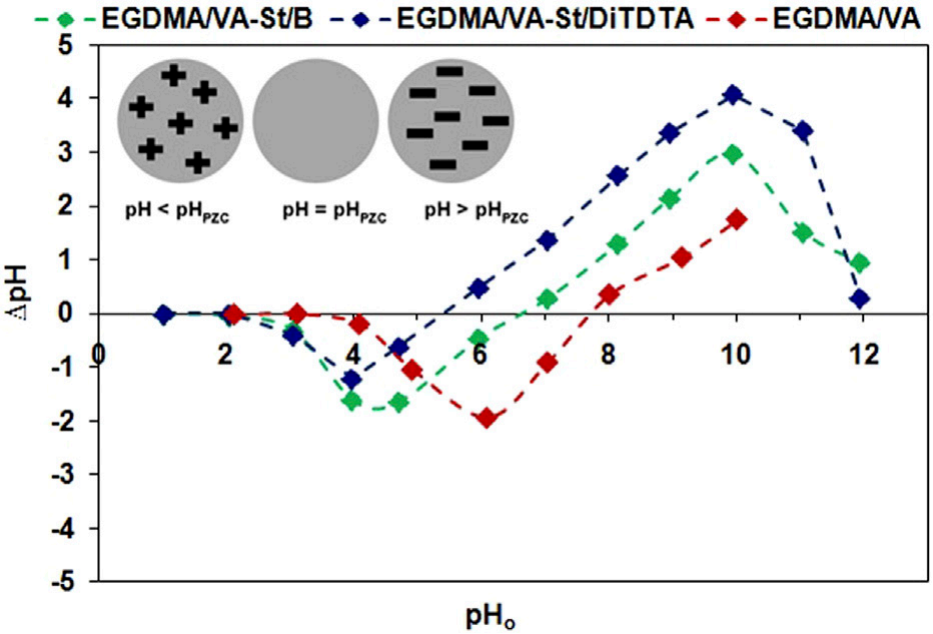
Point of zero charge of polymeric microspheres under discussion.

The point of zero charge for EGDMA/VA, EGDMA/VA-St/B, and EGDMA/VA-St/DiTDTA polymers was determined (Figure 8). This study showed that a higher pH_PZC_ was observed for EGDMA/VA (pH_PZC_ = 7.75) whereas for the polymers containing the modified starch pH_PZC_ = 6.62 and pH_PZC_ = 5.42 were found for EGDMA/VA-St/B and EGDMA/VA-St/DiTDTA, respectively. It was observed that the addition of modified starch causes a decrease in the point of zero charge of the polymers under discussion.

As mentioned previously, pH can also affect the metal species in aqueous solutions, which in turn can affect the adsorption efficiency. Taking into account Cu(II) (Yang et al., 2016), Ni(II) (Islam et al., 2019), and Zn(II) (El-Taib Heakal et al., 2018) species (percentage fraction of M(II) species *versus* pH) was observed that in all cases under discussion at strongly acidic solutions and up to about pH 6.5 for Cu(II) and Zn(II) as well as pH 8 for Ni(II) these metal exist in 100% in the form of divalent cations. After further increasing of pH hydroxide forms of positive, neutral or negative charage starts to form and starts to play a more significant role. The plot of M(II) species fraction (0%–100%, where 0.6 means 60%, 1 means 100%) depending on pH in the solutions containing 10 mg M(II)/L was obtained using Hydra and Medusa software (Supplementary Figure S1). The effect of pH on M(II) sorption on new polymeric microspheres was carried out. As was found (Supplementary Figure S2) pH influences the M(II) adsorption abd could be an important variable governing in their adsorption. The solution pH influences M(II) sorption onto adsorbent active sites due to the competition between the metal ions and H^+^ ions (Lakshmipathy and Sarada, 2015; Mensah et al., 2021). The highest percentage removal was obtained at pH 8 for all M(II) on EGDMA/VA, EGDMA/VA-St/B and EGDMA/VA-St/DiTDTA microspheres: Cu (%R ≥ 99.6), Ni(II) (%R ≥ 36.8) and Zn(II) (%R ≥ 60.9). As was reported in the literature with the increasing pH the heavy metals have tendency to precipitation and form hydroxides therefore the pH effect were conducted in the range up to pH 8. At strongly acidic solutions due to the functional groups protonation (H^+^ ions occupy most of the adsorption sites) the M(II) adsorption is usualy low as a concequence of electrostatic repulsion forces between H^+^ ions and the cationic forms of metal. The increase in M(II) sorption with increasing pH can be explained in terms of point of zero charge of the adsorbent and metal speciation occurring in the solution. At pH > pH_PZC_, the surface charge of the EGDMA/VA, EGDMA/VA-St/B and EGDMA/VA-St/DiTDTA microspheres is negative and hence positively charged Cu(II), Ni(II) and Zn(II) ions are adsorbed by the electrostatic attraction. When the pH value increases, the surface charge of the polymeric microspheres is more negatively charged therefore M(II) adsorption increased (Lakshmipathy and Sarada, 2015). For example, the percentage removal of Cu(II) increase from 60.1 (pH 6) throught 96.9 (pH 7) to 99.9 (pH 8). As a optimum pH, pH 6 was chosen for future studies to avoid the formation of soluble hydroxyl metal complexes (Mensah et al., 2021). For example, in the range of 7–12 precipitation of copper(II) hydroxide occurs wheras the precipitation of the amphoteric zinc(II) hydroxide starts at pH 8 (Podkościelna et al., 2019). As was mentioned by Guo et al. (2006) beyond pH 6.0 the Cu(II) adsorption on water-insoluble starch phosphate carbamate becomes masked by Cu(OH)_2_ precipitation therefore, both adsorption and precipitation jointly contribute to Cu(II) ion removal from solution. A selected optimum pH is in line with the literature (Wang and Guo, 2023).

Acid dyes such as AG16, which are salts of sulphonic acids, are strong electrolytes and dissociate in aqueous baths into coloured anions and sodium cations. Basic (cationic) dyes dissociate into coloured cations and chloride anions. Taking into account the pH_PZC_ of the EGDMA/VA (pH_PZC_ = 7.75) EGDMA/VA-St/B (pH_PZC_ = 6.62) and EGDMA/VA-St/DiTDTA (pH_PZC_ = 5.42) and the maximum adsorption of the dyes, the solutions of AG16 and BB3 with pH 4.5 and 8.8, respectively, were prepared for further studies. Such conditions provide the possibility of the electrostatic interactions between the negatively/positively charged surface of the adsorbents and the cationic/anionic forms of the dyes.

#### 3.2.2 Equilibrium studies

The amount of dyes or metal ions adsorbed at equilibrium by an adsorbent, known as the sorption capacity, is a quantity that characterizes the adsorptive material and allows an assessment of its applicability in industrial-scale wastewater treatment technologies. The results obtained are interpreted based on popular adsorption isotherm models, which provide insight into the nature of the interactions between adsorbent and adsorbate. The most popular equations describing adsorption in the liquid-solid system at equilibrium include Langmuir (Equation 4), Freundlich (Equation 5), Temkin (Equation 6), and Dubinin-Raduschkevich (Equation 7). The linear forms of these above models can be expressed by the following formulas:

$$\frac{C_{e}}{q_{e}}=\frac{1}{Q_{0}k_{L}}+\frac{C_{e}}{Q_{0}}$$ (4)

$$\log\;q_{e}=\log\;k_{F}+\frac{1}{n}\log\;C_{e}$$ (5)

$$q_{e}=\left(\frac{RT}{b_{T}}\right)\ln A+\left(\frac{RT}{b_{T}}\right)\ln C_{e}$$ (6)

$$\ln q_{e}=\ln q_{m}-k_{DR}\varepsilon^{2}$$ (7)

where q_e_ (mg/g) is the adsorption capacity; C_e_ (mg/L) is the equilibrium concentration of dyes or metal ions in solutions, Q_0_ (mg/g) is the monolayer adsorption capacity; k_L_ (L/mg) is the Langmuir constant (relating to the free energy of adsorption); k_F_ (mg^1–1/n^ L^1/n^/g) and 1/n are the Freundlich constants regarding adsorption capacity and the surface heterogeneity, respectively; R (J/mol K) is gas constant, T (K) is temperature; A (L/mg) and b_T_ (J/mol g/mg) are the Temkin constants; q_m_ (mg/g) is maximum adsorption capacity; k_DR_ (mol^2^/J^2^) is constant concerning the adsorption energy; ε (J/mol) is adsorption potential (Murphy et al., 2023).

According to the Langmuir adsorption model, retention occurs at certain energetically homogeneous sites contained within the adsorbent and is monolayer in nature, with no lateral interactions and steric hindrance among adsorbates. These conditions are hardly ever met and are the main weaknesses of this model. Nevertheless, the model is still a popular method for describing adsorption equilibrium, mainly due to its simplicity in solution. In contrast to this model, the Freundlich isotherm describes the non-ideal and multilayer adsorption of solute on the adsorbent surface. The Freundlich model is not thermodynamically consistent and does not approach Henry’s law at low concentrations, which may lead to overestimation or underestimation of equilibrium capacity. It also has no saturation limit at high concentrations. The Temkin isotherm model predicts that adsorption is heterogeneous and multilayered. This model disregards both the very high and extremely low concentration of adsorbate. It also takes into consideration the influence of indirect interactions between the adsorbate and the adsorbent on the heat of adsorption of the adsorbed molecules in the layer, which decreases linearly rather than logarithmically (as in the case of Freundlich’s model) with increasing solid surface coverage. Dubinin-Raduschkevich isotherm model assumed that the adsorption process was related to filling the volume of the micropores, as opposed to layer-by-layer adsorption on the pore walls. In addition, the Dubinin-Raduschkevich isotherm model can be used to estimate the mean free energy E (E = 1/ $\sqrt{2k_{DR}}$ ), which can distinguish the type of adsorption process: physical, or chemical. The Dubinin-Raduschkevich model successfully predicts equilibrium adsorption capacities at intermediate and high concentrations by pore filling mechanism, however, it fails to approach Henry’s law at low concentration levels (Laskar and Hashisho, 2020).

The parameter of above mentioned isotherms was calculated from the graphs: C_e_/q_e_ vs. C_e_, log q_e_ vs. log C_e_, q_e_ vs. ln C_e,_ and ln q_e_ vs. ε^2^ applying linear regression and are listed in Table 3.

**TABLE 3:** Isotherm parameters for dyes and heavy metal ions sorption on EGDMA/VA, EGDMA/VA-St/B, and EGDMA/VA-St/DiTDTA.

Isotherm	Parameters	EGDMA/VA	EGDMA/VA-St/B	EGDMA/VA-St/DiTDTA
AG16				
Freundlich	k_F_ (mg^1-1/n^L^1/n^/g) 1/n *R* ^2^	0.896 0.710 0.998	1.2 0.697 0.986	8.9 0.479 0.988
Langmuir	k_L_ (L/mg) Q_0_ (mg/g) *R* ^2^	0.013 42.2 0.889	0.014 51.8 0.738	0.170 57.9 0.967
Temkin	b_T_ (J/mol g/mg) A (L/mg) *R* ^2^	406.5 0.319 0.867	350.2 0.399 0.776	319.1 6.6 0.903
Dubinin-Radushkevich	q_m_ (mg/g) k_DR_ (mol^2^/J^2^) E (kJ/mol) *R* ^2^	10.0 1.67 · 10^−8^ 0.55 0.491	11.1 8.4 · 10^−7^ 0.77 0.435	23.1 6.4 · 10^−8^ 2.8 0.714
BB3				
Freundlich	k_F_ (mg^1-1/n^L^1/n^/g) 1/n *R* ^2^	29.9 0.614 0.993	31.0 0.512 0.968	33.8 0.683 0.978
Langmuir	k_L_ (L/mg) Q_0_ (mg/g) *R* ^2^	0.216 200.8 0.852	0.166 209.5 0.749	0.177 256.9 0.811
Temkin	b_T_ (J/mol g/mg) A (L/mg) *R* ^2^	107.1 14.9 0.774	136.0 32.9 0.678	97.1 14.0 0.740
Dubinin-Radushkevich	q_m_ (mg/g) k_DR_ (mol^2^/J^2^) E (kJ/mol) *R* ^2^	59.9 3.4 · 10^−8^ 3.9 0.690	59.9 2.2 · 10^−8^ 4.8 0.707	57.8 3.25 · 10^−8^ 3.9 0.651
Cu(II)				
Freundlich	k_F_ (mg^1-1/n^L^1/n^/g)	2.5	3.6	3.1
	1/n	0.390	0.562	0.486
	*R* ^2^	0.996	0.992	0.995
Langmuir	k_L_ (L/mg)	0.013	0.023	0.020
	Q_0_ (mg/g)	113.7	88.1	90.7
	*R* ^2^	0.808	0.872	0.891
Temkin	b_T_ (J/mol g/mg)	157.9	182.0	175.2
	A (L/mg)	0.376	0.580	0.469
	*R* ^2^	0.862	0.864	0.879
Dubinin-Radushkevich	q_m_ (mg/g)	32.5	31.7	31.7
	k_DR_ (mol^2^/J^2^)	1.9 · 10^−6^	8.3 · 10^−7^	1.3 · 10^−6^
	E (kJ/mol)	513.7	777.0	629.1
	*R* ^2^	0.606	0.563	0.586
Ni(II)				
Freundlich	k_F_ (mg^1-1/n^L^1/n^/g)	1.6	1.7	1.2
	1/n	0.819	0.803	0.862
	*R* ^2^	0.997	0.996	0.990
Langmuir	k_L_ (L/mg)	0.008	0.009	0.005
	Q_0_ (mg/g)	143.4	136.1	179.7
	*R* ^2^	0.951	0.933	0.802
Temkin	b_T_ (J/mol g/mg)	137.6	140.4	135.3
	A (L/mg)	0.253	0.265	0.212
	*R* ^2^	0.911	0.907	0.904
Dubinin-Radushkevich	q_m_ (mg/g)	36.9	36.8	37.6
	k_DR_ (mol^2^/J^2^)	4.9 · 10^−6^	4.5 · 10^−6^	7.8 · 10^−6^
	E (kJ/mol)	318.1	332.2	252.8
	*R* ^2^	0.717	0.714	0.776
Zn(II)				
Freundlich	k_F_ (mg^1-1/n^L^1/n^/g)	1.4	1.6	1.5
	1/n	0.870	0.849	0.834
	*R* ^2^	0.985	0.958	0.991
Langmuir	k_L_ (L/mg)	0.007	0.010	0.007
	Q_0_ (mg/g)	176.6	142.0	158.8
	*R* ^2^	0.802	0.820	0.864
Temkin	b_T_ (J/mol g/mg)	126.4	126.3	133.9
	A (L/mg)	0.259	0.294	0.276
	*R* ^2^	0.890	0.936	0.878
Dubinin-Radushkevich	q_m_ (mg/g)	38.6	39.0	35.1
	k_DR_ (mol^2^/J^2^)	4.7 · 10^−6^	4.0 · 10^−6^	3.2 · 10^−6^
	E (kJ/mol)	327.4	355.1	397.5
	*R* ^2^	0.731	0.708	0.635

Values of the determination coefficients (*R* ^2^) were calculated and taken into account to determine the best-fitting model for the adsorption data (Murphy et al., 2023). The fitting of experimental equilibrium date to the isotherm models for dyes and metal ions are presented in Supplementary Figure S3. The analysis of the calculated isotherm parameters summarised in Table 3 allows the conclusion that the Freundlich adsorption model can be applied to describe the adsorption of both dyes (AG16 and BB3) and heavy metal ions (Cu(II), Ni(II) and Zn(II)). The highest determination coefficients *R* ^2^ of the experimental data to the Freundlich model were obtained during the adsorption of dyes and heavy metal ions on the EGDMA/VA, EGDMA/VA-St/B and EGDMA/VA-St/DiTDTA. They were in range of 0.986–0.998, 0.968–0.993, 0.992–0.996,0.990–0.997 and 0.958–0.991 for AG16, BB3, Cu(II), Ni(II) and Zn(II), respectively. 1/n values are smaller than 1, which means that the adsorption conditions are favourable for the dyes and metal ions removal by the EGDMA/VA, EGDMA/VA-St/B and EGDMA/VA-St/DiTDTA and is of a physical nature. Supplementary Figure S4 shows the ATR/FT-IR spectra after sorption of dyes (BB3, AG16) and Cu(II) ions. Compared to the initial materials, no major changes were observed in the spectra, only slight differences in band intensities can be seen, proving that the physical interaction rather than the chemical one takes place. The values of the *R* ^2^ for the Langmuir, Temkin and Dubinin-Raduschkevich isotherm models were significantly lower which indicates that they cannot be used to describe the experimental data obtained. The largest values of the k_F_ coefficient were calculated for BB3 and they were equalled to 29.9 mg^1-1/n^L^1/n^/g for EGDMA/VA, 31.0 mg^1-1/n^L^1/n^/g for EGDMA/VA-St/B and 33.8 mg^1-1/n^L^1/n^/g for EGDMA/VA-St/DiTDTA. Considerng the values of k_F_ (expressed as mg^1-1/n^L^1/n^/g), the following affinity series of removed dyes and metal ions can be presented:

BB3 (29.9) > Cu(II) (2.5) > Ni(II) (1.6) > Zn(II) (1.4) > AG16 (0.9) for EGDMA/VA.

BB3 (31) > Cu(II) (3.6) > Ni(II) (1.7) >Zn(II) (1.6) > AG16 (1.2) for EGDMA/VA-St/B.

BB3 (33.8) > AG16 (8.9) > Cu(II) (3.1) > Zn(II) (1.5) > Ni(II) (1.2) for EGDMA/VA-St/DiTDTA.

The applicability of the adsorbents in the treatment of wastewaters from dyes and metal ions can be assessed by comparing the experimental data obtained with those available in the literature. The summary presented in Table 4 allows an overview of the adsorbents that were used for removal of Cu(II), Ni(II), Zn(II), BB3 and AG16 and a comparison of the sorption capacities obtained. While starch-containing adsorbents are used for the sorption of Cu(II), Ni(II), and Zn(II), they are not popular for the removal of dyes such as AG16 and BB3. Thus, the results presented here make a valuable contribution to the applicability of adsorbents containing bioadditives such as starch for the treatment of textile industry wastewater containing AG16 and BB3.

**TABLE 4:** Adsorbents for dyes and heavy metal ions removal–literature review.

Adsorbent based on starch	Metal ions/dyes	Adsorption capacity (mg/g)	References
Walnut shell ash/starch/iron oxide (Fe_3_O_4_)	Cu(II)	45.4	Foroutan et al. (2022)
Starch/Fe_3_O_4_-g-p (AA-r-HEMA)	Cu(II)	75.5	Hou et al. (2021)
Magnetic starch-g-polyamidoxime/montmorillonite/Fe_3_O_4_ nanocomposites	Cu(II)	163	Mahdavinia et al. (2016)
Silica-sand/anionized-starch composite	Cu(II)	383.08 ± 13.5	Li et al. (2020)
Carboxymethyl starch-g-polyvinyl imidazole	Cu(II)	83.6	Sekhavat Pour and Ghaemy (2015)
Starch–chitosan-based hydrogel microspheres	Cu(II)	47.87	Li et al. (2021a)
Crosslinked carboxymethyl sago starch/citric acid hydrogel	Cu(II)	36.56	Keirudin et al. (2020)
Three-dimensional nanoporous starch-based nanomaterial functionalized by EDTA (EDTA/3D-PSN))	Cu(II)	354.15	Fang et al. (2020)
Starch nanocrystals (SNCs)	Cu(II)	35.83	Chen et al. (2022)
EGDMA/VA EGDMA/VA-St/B EGDMA/VA-St/DiTDTA	Cu(II)	61.01 61.61 63.66	This study
Starch–chitosan-based hydrogel microspheres	Ni(II)	27.18	Li et al. (2021b)
Crosslinked carboxymethyl sago starch/citric acid hydrogel	Ni(II)	16.21	Keirudin et al. (2020)
Starch-g-polyacrylamide/Fe_3_O_4_/graphene oxide nanocomposite	Ni(II)	290	Keirudin et al. (2020), Khoo et al. (2023)
Native corn starch (NS)	Ni(II)	8.29	Soto et al. (2015)
Itaconate starch semiester (SI)	Ni(II)	6.38	
Itaconate starch diester (DI)	Ni(II)	5.1	
EGDMA/VA EGDMA/VA-St/B EGDMA/VA-St/DiTDTA	Zn(II)	82.88 81.57 84.25	This study
Crosslinked carboxymethyl sago starch/citric acid hydrogel	Zn(II)	18.45	Keirudin et al. (2020)
Starch nanocrystals (SNCs)	Zn(II)	46.13	Chen et al. (2022)
Native corn starch (NS)	Zn(II)	3.18	Soto et al. (2015)
Itaconate starch semiester (SI)	Zn(II)	7.84	
Itaconate starch diester (DI)	Zn(II)	8.44	
EGDMA/VA EGDMA/VA-St/B EGDMA/VA-St/DiTDTA	Ni(II)	68.95 68.64 67.94	This study
Magnetic geopolymer/Fe_3_O_4_ composite	AG16	400	Rossatto et al. (2020)
Activated carbon based on rice bran	AG16	1.05–1.36	Sankar et al. (1999)
Molecularly imprinted polymers (MIP)	AG16	6.9	Foguel et al. (2017)
Adsorbent from volcanic rock powder waste	AG16	49.1	Rossatto et al. (2023)
Anion exchange resin Lewatit S 6368 A	AG16	625.2–811.6	Wrzesińska et al. (2021)
EGDMA/VA EGDMA/VA-St/B EGDMA/VA-St/DiTDTA	AG16	29 39 56	This study
Starch/acrylonitrile-amidoxime	BB3	6.6–9.1	Abdel-Aal et al. (2006)
Adsorbent obtained from the stem of the *Silybum Marianum* plant	BB3	13.96–36.81	Börklü Budak (2023)
Polymeric adsorbent with phenylvinylphosphine oxide	BB3	32.3	Wawrzkiewicz et al. (2023)
Molecularly imprinting polymer	BB3	78.13–91.74	Sadia et al. (2022)
Pd–Ni nanoparticles supported on activated carbon	BB3	333	Alam et al. (2021)
EGDMA/VA EGDMA/VA-St/B EGDMA/VA-St/DiTDTA	BB3	193 190 194	This study

#### 3.2.3 Kinetic studies

**FIGURE 9:**
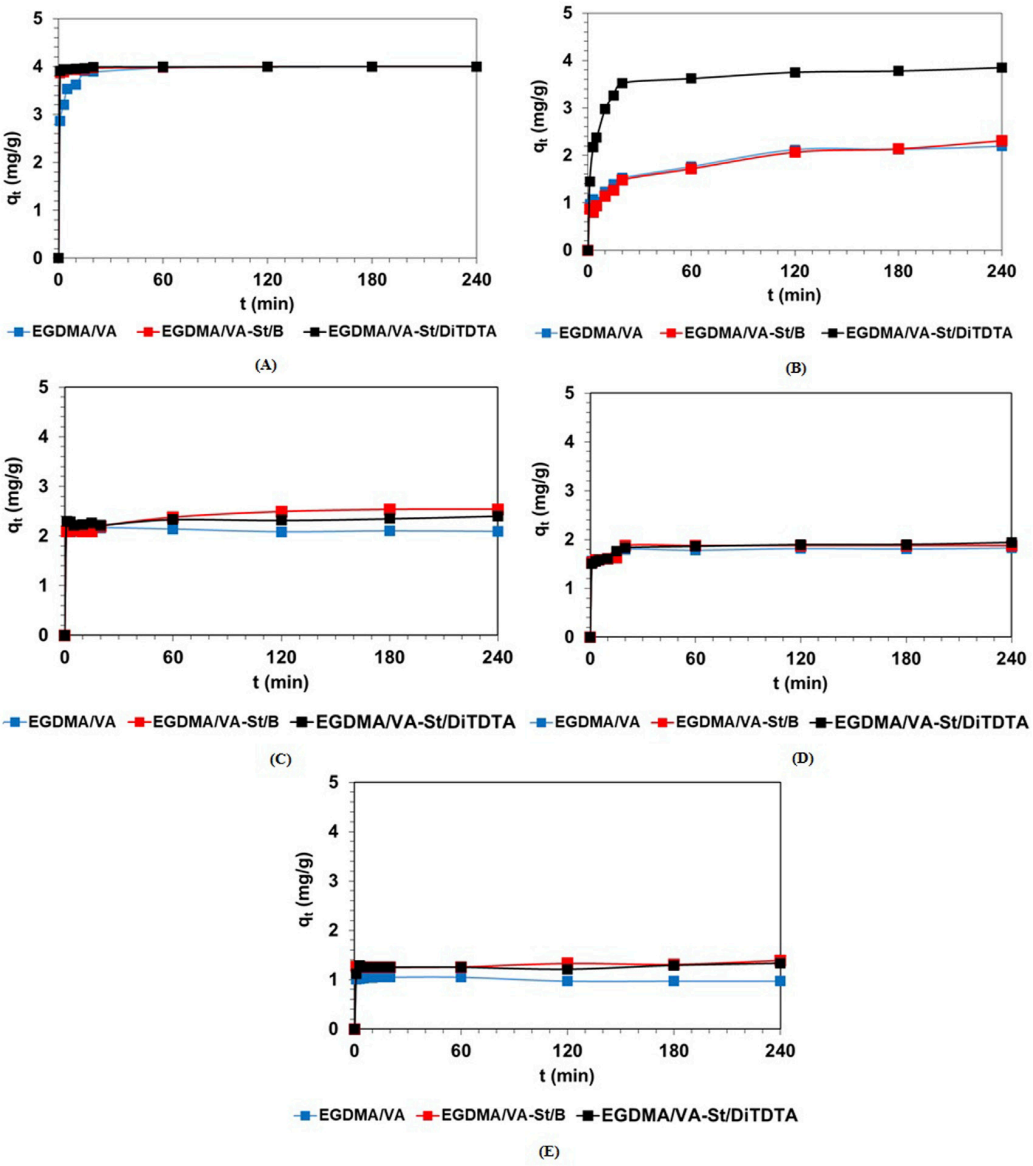
Time impact on **(A)** BB3, **(B)** AG16, **(C)** Cu(II), **(D)** Ni(II), **(E)** Zn(II) adsorption by the EGDMA/VA, EGDMA/VA-St/B and EGDMA/VA-St/DiTDTA.

An important aspect of studying adsorption and understanding the mechanism of adsorption is to analyze the time required to fully saturate the adsorbent surface with adsorbate molecules. Figure 9 illustrates the impact of contact time on the amount of BB3, AG16 and Cu(II), Ni(II) and Zn(II) sorbed by the EGDMA/VA, EGDMA/VA-St/B and EGDMA/VA-St/DiTDTA.

Adsorption of BB3 dye (Figure 9A) and M(II) ions (Figures 9C–E) on EGDMA/VA, EGDMA/VA-St/B, and EGDMA/VA-St/DiTDTA are very fast, equilibrium is reached in less than 20 min for M(II) ions and 40 min for BB3 of phase contact in the solution of the initial dye or heavy metal concentration 10 mg/L regardless of the type of adsorbent used. It can be observed that the surface adsorption sites become occupied by the BB3 and metal cations and no diffusion into the pores takes place. The q_e_ values for BB3 are found to be 4 mg/g whereas for M(II) ions being in the range from 0.97 to 2.5 mg/g for EGDMA/VA, EGDMA/VA-St/B and EGDMA/VA-St/DiTDTA. In the case of M(II) ions adsorption, the q_e_ values are the highest for EGDMA/VA-St/B being 2.5 mg/g for Cu(II) > 2.0 mg/g for Ni(II) > 1.4 mg/g for Zn(II) (EGDMA/VA-St/B ≈ EGDMA/VA-St/DiTDTA > EGDMA/VA).

As was reported in the literature, high heavy metal adsorption rates were observed at the beginning of the adsorption process and the adsorption equilibrium was reached rapidly (Dam and Kim, 2009; Miao et al., 2011; Podkościelna et al., 2019). For example, during the sorption of Cu(II), and Ni(II) ions on the surface-imprinted core-shell type polymethacrylate microspheres, the equilibrium was reached within 30 min (Dam and Kim, 2009). The amount of Cu(II), Ni(II), and Zn(II) adsorbed on the styrene with divinylbenzene microspheres modified by–SH groups at the experimental conditions such as the initial concentration 0.001–0.003 mol/L, weight of 0.1 g, shaking speed 180 rpm, temperature of 293 K, pH 5 increases with time and reaches a maximum value at about 90 min of phase contact time (Podkościelna et al., 2019).

According to Alam et al. (2024) adsorption of BB3 from the solution of the concentration 0.001 M at 283–303 K reached equilibrium after 10 min of sorption using hydrogels containing copper nanoparticles. 20 min of phase contact was adequate to reach equilibrium during the sorption of BB3 of the concentrations 0.05–0.1 g/L at pH 8 by the hydromagnesite stromatolite (Karakuş et al., 2020). Börklü Budak (2023) reported that BB3 (10 mg/L) adsorption on the 0.2 g of silybum marianum stem as a low-cost adsorbent resulted in 86.09% and q_e_ = 0.43 mg/g at 30 min. After carbonization of silybum marianum stem at 1073 K BB3 (15 mg/L) removal was found to be 99.99% and q_e_ = 0.99 mg/g at 40 min. Applying the starch-amidoxime adsorbent in BB3 (100 mg/L, pH 4–7) removal for more than 10 days was required to reach equilibrium (Abdel-Aal et al., 2006).

Adsorption of AG16 occurred much more slowly, in the initial sorption time the distribution of experimental points is not as steep as in the case of BB3 (Figure 9B). A gradual retention of AG16 by the adsorbents can be observed between 1 and 60 min for EGDMA/VA, EGDMA/VA-St/B, and up to 120 min for EGDMA/VA-St/DiTDTA. The equilibrium state of AG16 adsorption is established after 120 min of phase contact time and q_e_ was equaled to 2.2 mg/g for EGDMA/VA and 2.3 mg/g for EGDMA/VA-St/B. Starch modified by DiTDTA improves the adsorption properties of the prepared polymer based on EGDMA/VA and increased adsorption of AG16 on this material can be observed. The sorption capacity of EGDMA/VA-St/DiTDTA towards AG16 is 3.9 mg/g.

Fast adsorption of AG16 on the magnetic geopolymer/Fe_3_O_4_ composite prepared from metakaolin, biogenic rice husk silica, and magnetite within 30 min was reported by Rossatto et al. (2020). During the AG16 adsorption on the anion exchanger Lewatit S5528 of the strongly basic functional groups, the amount of dye adsorbed increased with the phase contact time increase (e.g., for 1 min q_t_ = 20.7 mg/L, whereas for 15 min q_t_ = 49.5 mg/L, initial AG16 concentration 500 mg/L) and reached equilibrium after 180 min (Wrzesińska et al., 2021).

Adsorption of M(II) ions and dyes demonstrates the rapid removal of these contaminants, which is very promising from the time-consuming and economic aspects of adsorption.

#### 3.2.4 Salt and surfactant impact

**FIGURE 10:**
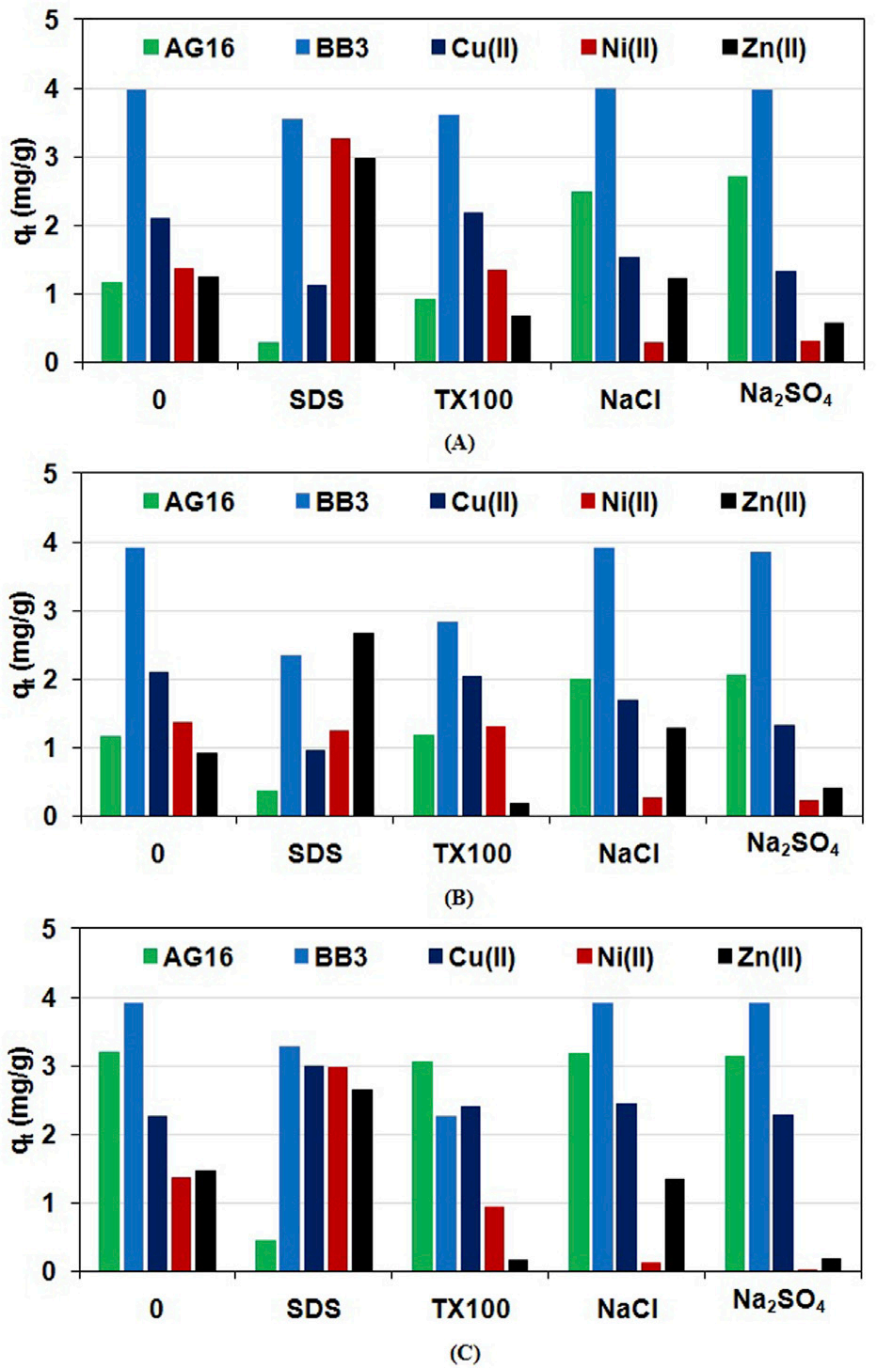
Sorption of dyes and metal ions in the presence of surfactants and electrolytes on the **(A)** EGDMA/VA, **(B)** EGDMA/VA-St/B and **(C)** EGDMA/VA-St/DiTDTA.

The presence of surfactants and salts in wastewater, especially from the textile industry, is unavoidable (Ardhan et al., 2022). These substances are added in significant quantities to dyeing baths to clean the fibre prior to the dyeing process and also enable it to bind with the coloured material. They enter the wastewater from the dyeing baths in the same quantities as they are added initially. Their presence in solution can alter sorption capacities significantly compared to aqueous solutions without these additives. It is therefore extremely important to evaluate their presence in the aqueous environment on the sorption efficiency of dyes or metal ions removal. Such a study was carried out by assessing the adsorption (q_t_ at t = 15 min) of the AG16, BB3, Cu(II), Ni(II), and Zn(II) at the concentrations of 10 mg/L in the presence of surfactants (0.5 g/L SDS or TX100) and electrolytes (5 g/L NaCl or Na_2_SO_4_). The presence of NaCl and Na_2_SO_4_ did not influence the adsorption of BB3 on the EGDMA/VA, EGDMA/VA-St/B, and EGDMA/VA-St/DiTDTA while anionic SDS and nonionic TX100 surfactants reduced the BB3 adsorption on the synthesized adsorbents as presented in Figure 10.

AG16 uptake by the EGDMA/VA, EGDMA/VA-St/B, and EGDMA/VA-St/DiTDTA dropped in the presence of SDS. The addition of the TX100 did not influence the retention of AG16 on the adsorbents. No impact of the electrolytes was observed on the AG16 sorption by the EGDMA/VA-St/DiTDTA while an increase in dye adsorption was noticed on the EGDMA/VA and EGDMA/VA-St/B. The increase in sorption capacity may be due to the disaggregation of dyes in aqueous solution in the presence of electrolytes, which is a well-known phenomenon (Dzieniszewska, et al., 2020). The amount of ‘disaggregated’ cations then increases and there is enhanced adsorption in the solid phase, i.e., in the polymer microspheres. The reduction in the amount of dye adsorbed may be the result of competitive sorption, smaller cations, or a reduction in the concentration of dye in aqueous solution due to its interaction with surfactants. Increase or decrease the adsorption of dyes such as C.I. Basic Yellow 2, C.I. Basic Blue 3, and C.I. Basic Red 46 on the co-participated lignin hybrids and carbon-silica composite in the presence of electrolytes and surfactants was previously described by Wawrzkiewicz et al. (2020, 2025) and Wiśniewska et al. (2022).

The Cu(II), Ni(II) and Zn(II) uptake by the EGDMA/VA, EGDMA/VA-St/B, and EGDMA/VA-St/DiTDTA in the presence of Na_2_SO_4_ and NaCl electrolytes usually decreased. No effect on the metal uptake was observed for Cu(II) adsorption in the presence of Na_2_SO_4_ on the EGDMA/VA-St/DiTDTA adsorbent and in the systems containing Zn(II) in the presence of NaCl for all adsorbents. The addition of the TX100 did not affect Cu(II) and Ni(II) adsorption and decreased Zn(II) retention on all adsorbents. The presence of the SDS generally increased the Cu(II), Ni(II), and Zn(II) uptake except for the EGDMA/VA + Cu(II) and EGDMA/VA-St/B + Cu(II) systems (decrease). The decrease in the sorption capacity of heavy metals in the presence of the electrolytes may be related to the competitive interactions between the ions introduced during the electrolyte addition and the adsorbate ions of the adsorbent active sites (Han et al., 2006). The SO_4_^2-^ or Cl^−^ ions can form anionic complexes with metal cations and change the nature of the interactions depending on the charge of the adsorbent surface (Kołodyńska et al., 2017). For example, from the attraction between Cu(II) cations and the negatively charged surface of the adsorbent to repulsion between anionic copper complexes and the positively charged surface. Similar results, which mean reduction of the copper adsorption capacities in the presence of NO_3_^−^, SO_4_^2-^ or Cl^−^ interfering ions, were previously observed for Lewatit FO 36, Purolite Arsen X^np^, W2 KPS materials (Kołodyńska et al., 2015; Kołodyńska et al., 2017). As was reported in the literature the effect of non-ionic and ionic surfactants on changes in M(II) adsorption capacities of various materials are diversified. During the sorption process of metal in the presence of surfactants, there could proceeds simultaneous sorption of metal as well as a surfactant in unbound form, as well as in metal-surfactant bound and the speed of the sorption process could be determined by the inter-particle diffusion (Kaušpėdienė et al., 2007; Wołowicz et al., 2023).

### 3.3 Desorption studies

The ability to regenerate the adsorbent makes the purification process economical and allows the adsorbent to be reused (Bayuo et al., 2024). At the same time, it reduces the generation of large quantities of by-products and waste into the wastewater (Rápó and Tonk, 2021). Reagents should be selected in such a way that they do not further pollute the aquatic environment (Bayuo et al., 2024; Sahu and Poler, 2024). Moreover, during eluting agent selection the desorption performance, economic viability, and non-hazardous influence on the adsorbent should be in mind (Bayuo et al., 2024). Aqueous solutions of 1 M NaCl, 1 M HCl, and 1 M NaOH and their mixtures with 50% v/v methanol were used as eluents in dyes desorption studies whereas in the systems containing Cu(II), Ni(II) or Zn(II) ions 1 M HCl, 1 M HNO_3_, 1 M H_2_SO_4_, 1 M NH_3_·H_2_O, and 1 M NaOH solutions were applied. There may be some environmental controversy surrounding the use of methanol, but it can be degraded to carbon dioxide and water by specialized bacterial cultures, as confirmed by the Kaszycki and Koloczek (2002), Kaszycki et al. (2006) studies.

The desorption efficiency of dyes or heavy metals is diverse and depends on many conditions such as pH of the desorption agent, desorption time, concentration of desorption agent, stirring speed, volume of the desorption agent used, temperature of eluent solution (Bayuo et al., 2024).

**FIGURE 11:**
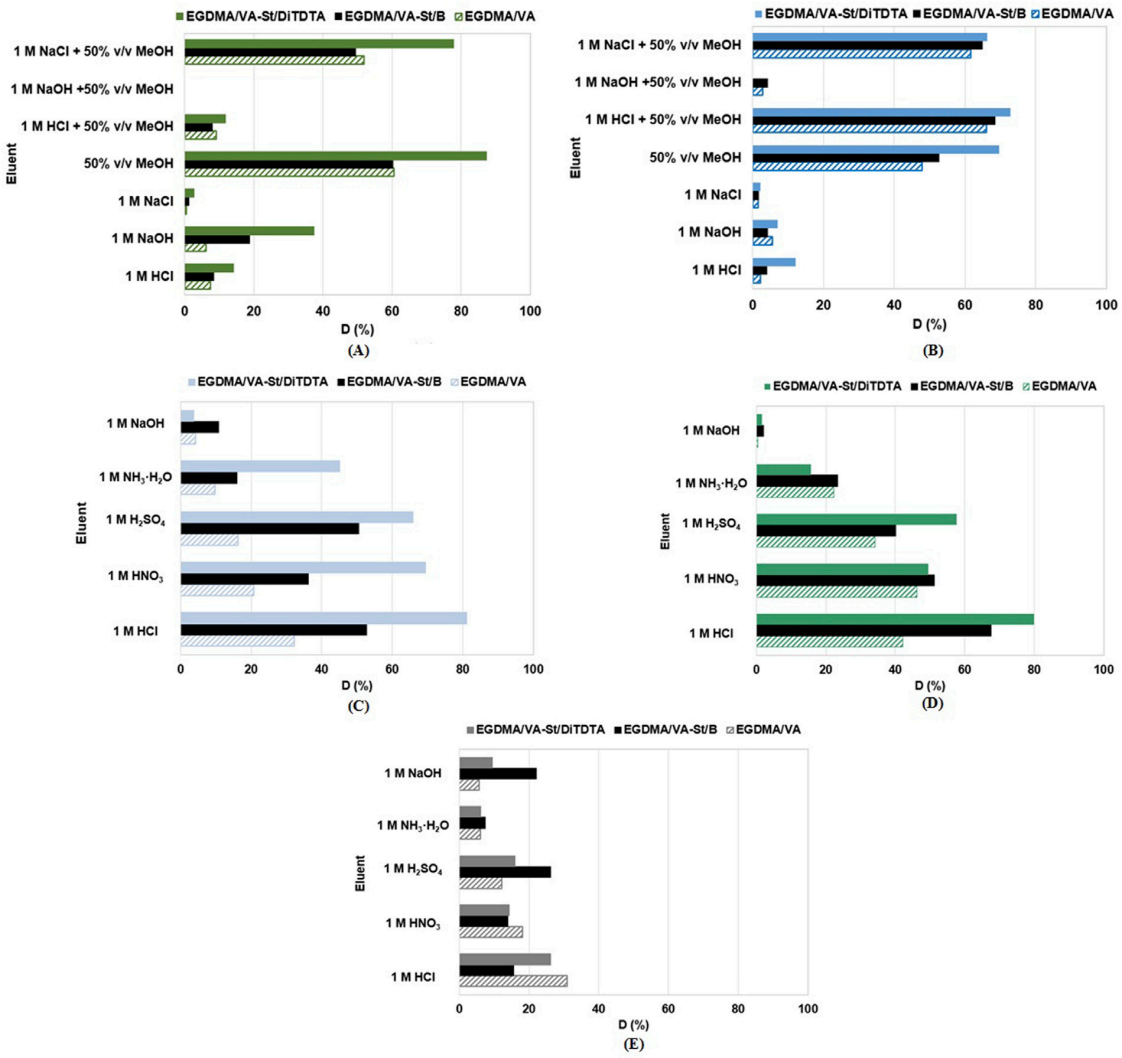
Desorption of **(A)** AG16, **(B)** BB3 and **(C)** Cu(II), **(D)** Ni(II), **(E)** Zn(II) from EGDMA/VA, EGDMA/VA-St/B and EGDMA/VA-St/DiTDTA.

Aqueous solutions of 1 M HCl, 1 M NaCl, and 1 M NaOH were not effective in leaching adsorbed AG16 and BB3 from the adsorbent phase as the percentage of desorption did not exceed a value of 38% (Figure 11). 50% v/v methanol desorbed BB3 at 48%, 53%, 70%, and AG16 at 61%, 60%, and 87% from EGDMA/VA, EGDMA/VA-St/B and EGDMA/VA-St/DiTDTA, respectively. The addition of 50% v/v methanol to 1 M NaCl significantly increased the desorption efficiency of dyes from adsorbents containing modified starch. A similar increase in desorption efficiency of basic dyes such as BY2 (C.I. Basic Yellow 2) and BB3 from adsorbents containing modified lignin and starch was described by Wawrzkiewicz et al. (2022, 2025). The effectiveness of methanol in removing dyes from the adsorbent phase confirms the physical nature of the interaction in the adsorbent/adsorbate system. According to research carried out by Jiang et al. (2023) increase in desorption of dyes such as methylene blue, Congo red, methyl orange, and methyl green from the composite hydrogels containing β-cyclodextrin, acrylamide, and acrylic acid was observed using 75% ethanol solution, with small amount of acids (acetic, citric, salicylic and oxalic) added.

Heavy metals desorption efficiency from EGDMA/VA, EGDMA/VA-St/B, and EGDMA/VA-St/DiTDTA is diversified and depends on the desorption agent type, adsorbate and the properties of adsorbents. As can be seen from Figure 11C, the desorption of Cu(II) using 1 M NaOH and 1 M NH_3_·H_2_O was not effective enough and was not more than 10.8% and 45.1%, respectively. The desorption of Cu(II) was better when the acids were used as the eluting agents. The best desorption yield was obtained using the 1 M HCl. The desorption efficiencies, in this case, were 81.2%, 52.8%, and 32.2% for EGDMA/VA-St/DiTDTA, EGDMA/VA-St/B and EGDMA/VA, respectively. The desorption studies also reflect to the strength of the interactions between the adsorbate and the adsorbents. The desorption efficiency of Cu(II) was usually much higher for EGDMA/VA-St/DiTDTA compared to EGDMA/VA-St/B and EGDMA/VA, indicating weaker interactions in this case. Similar trends as in the case of systems containing Cu(II) were also observed during Ni(II) desorption (D = 0.4–2.1% - 1 M NaCl; D = 15.8–23.3% - 1 M NH_3_·H_2_O, 34.2%–57.5% - 1 M H_2_SO_4_, 46.3%–51.2% - 1 M HNO_3_). The greatest desorption of Ni(II) was also observed with 1 M HCl (79.9% for EGDMA/VA-St/DiTDTA >67.7% for EGDMA/VA-St/B > 42.1% for EGDMA/VA). The desorption efficiency of Zn(II) was low in all cases (below 31%) and much lower compared to Cu(II) and Ni(II) even with the application of 1 M HCl solutions (D = 15.6–31.0%). The successful desorption of heavy metals using acidic eluents such as H_2_SO_4_, HCl, HNO_3,_ and H_3_PO_4_ have been previously proved in the literature (Bayuo et al., 2024). Furthermore, the chelating EDTA can desorb retained heavy metals (El-Denglawey et al., 2022) similar to the alkaline solutions from which sodium carbonate, sodium hydroxide, and sodium bicarbonate have been shown good desorption ability in heavy metal ions recovery from exhausted adsorbents. Salts such as sodium chloride, sodium nitrate, magnesium sulfate, or calcium chloride could be also effective (Chatterjee and Abraham, 2019; Mishra, 2014). For example, Cu(II) retained on the cross-linked starch phosphate carbamates was effectively desorbed by using the 1 M HCl solutions with >96% efficiency due to the higher affinity of H^+^ ions to the active groups, thus releasing the Cu(II) ions (Guo et al., 2006). Although in some cases non-quantitative desorption is observed, in most cases the adsorbent could be reused and applied again in the next sorption-desorption cycle (Bayuo et al., 2024).

## 4 Conclusion

To address the challenges associated with the effective removal of inorganic and organic impurities by the adsorption technique, new adsorbents in the form of polymer microspheres (EGDMA/VA) containing starch modified with boric acid (EGDMA/VA-St/B) and dodecyl-S-thiuronium dodecylthioacetate (EGDMA/VA-St/DiTDTA) have been synthesized and then they were characterized by using the ATR/FT-IR, DSC, EDS, SEM, BET and pH_PZC_ methods. In the next step their pottential for dyes such as C.I. Basic Blue 3 (BB3) and C.I. Acid Green 16 (AG16) and heavy metal ions M(II): Cu(II), Ni(II), and Zn(II) removal from water and wastewater. The ATR/FT-IR and SEM analyses revealed the successful incorporation of the biocomponent into the polymer matrix, resulting in the formation of microspheres. The incorporation of the modified starch has been observed to enhance the thermal resistance of the EGDMA/VA-St/B and EGDMA/VA-St/DiTDTA adsorbents in comparison to the EGDMA/VA. Furthermore, the point of zero charge was observed to decrease from 7.75 (EGDMA/VA) through 6.62 (EGDMA/VA-St/B) to 5.42 (EGDMA/VA-St/DiTDTA), and a slight decrease in the specific surface area was noted, ranging from 207 m^2^/g through 184 to 169 m^2^/g, respectively. From an economic standpoint, the obtained polymeric microspheres demonstrate considerable potential for use in the adsorption of dyes and heavy metal ions. This is due to their high experimental adsorption capacities, with the highest obtained for the BB3 dye at a range of 193 mg/g to 194 mg/g across all adsorbents. The second advantage is the good adsorption kinetics, which ensure the rapid removal of dyes and heavy metal ions from the aqueous solutions within the first 20 min for M(II), 40 min for BB3, and 120 min for AG16. The values of q_t_ were equal to 4 mg/g for BB3, 2.2–3.9 mg/g for AG16 and 0.97–2.5 mg/g for M(II) at 10 mg/L initial concentration. Furthermore, the proposed adsorbents can be effectively regenerated through the use of 50% v/v methanol and its mixture with 1 M HCl, NaCl for dyes and 1 M HCl for M(II) ions and then be reused. The results demonstrate that the efficiency of adsorption in dye-adsorbent and M(II)-adsorbent systems is contingent upon the contact time between the phases, the initial concentration of the adsorbate and the presence of competing electrolytes, including NaCl, Na_2_SO_4_, and surfactants such as SDS and TX100. The dyes and metal ions interactions with the EGDMA/VA, EGDMA/VA-St/B, and EGDMA/VA-St/DiTDTA adsorption are rather physical because the Freundlich isotherm model described experimental data better than the Langmuir, Temkin, and Dubinin-Raduschkevich ones.

The results obtained in this study of preparation, physico-chemical characterization, and application of new polymeric adsorbents containing bicomponent in the environmental aspect are important from the cognitive and technological point of view. In addition, they may contribute to a significant improvement in the state of knowledge in this field and the development of new technological approaches to the treatment of wastewater of complex composition.

## Data Availability

The original contributions presented in the study are included in the article/Supplementary Material, further inquiries can be directed to the corresponding author.

## Abbreviations

AAS, Atomic Absorption Spectroscopy; AG16, C.I. Acid Green 16; AIBN, α,α′-azobis(isobutyronitrile); ASAP, Atmospheric Solids Analysis Probe; ATR/FT-IR, Attenuated Total Reflectance Fourier Transformed Infrared Spectroscopy; B, Boric acid; BB3, C.I. Basic Blue 3; BET, Brunauer-Emmett-Teller method; BJH, Barrett-Joyner-Halenda method; DiTDTA, Dodecyl-S-thiuronium dodecylthioacetate; DSC, Differential Scanning Calorimetry; EDS, Energy Dispersive X-ray Spectroscopy; PVA, Poly(vinyl alcohol); SDS, Sodium Dodecyl Sulfate (anionic surfactant); SEM, Scanning Electron Microscopy; St, Starch; TX100, t-octylphenoxypolyethoxyethanol, Triton X100 (non-ionic surfactant); VA, Vinyl acetate.
