# Supplementary material for: Polymeric resins containing modified starch as environmentally friendly adsorbents for dyes and metal ions removal from wastewater
Source: Front Chem. 2024 Oct 30;12:1496901. doi: 10.3389/fchem.2024.1496901 (PMC11557332; doi:10.3389/fchem.2024.1496901)
Supplement: Supplementary file 1 [file DataSheet1.docx]

Polymeric resins containing modified starch as environmentally friendly adsorbents for dyes and metal ions removal from wastewater

Anna Wołowicz^1*^, Monika Wawrzkiewicz^1^, Beata Podkościelna^2^, Bogdan Tarasiuk^2^,
Jadranka Blazhevska Gilev^3^, Olena Sevastyanova^4^

^1^Faculty of Chemistry, Institute of Chemical Sciences, Department of Inorganic Chemistry, Maria Curie-Sklodowska University in Lublin, Poland

^2^Faculty of Chemistry, Institute of Chemical Sciences, Department of Polymer Chemistry, Maria Curie-Sklodowska University in Lublin, Poland

^3^Faculty of Technology and Metallurgy, Ss. Cyril and Methodius University in Skopje, Skopje, R. N. Macedonia

^4^Wallenberg Wood Science Center, Department of Fibre and Polymer Technology, KTH Royal Institute of Technology, Stockholm, Sweden

*** Correspondence:**Anna Wołowicz
tel. +48 81 537 57 38, [anna.wolowicz@mail.umcs.pl](mailto:anna.wolowicz@mail.umcs.pl)

Supplementary Material

# Supplementary Figures


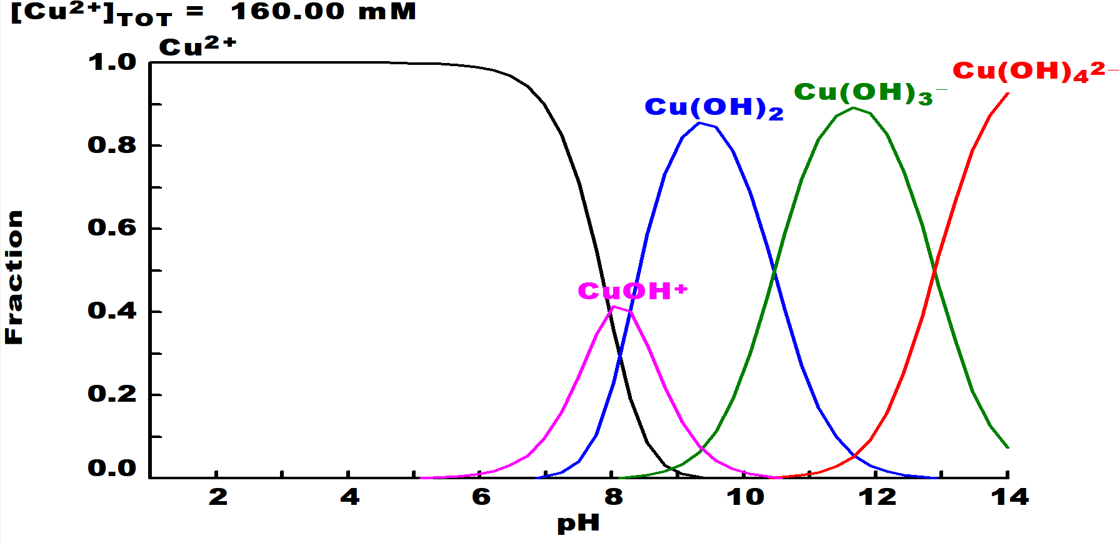

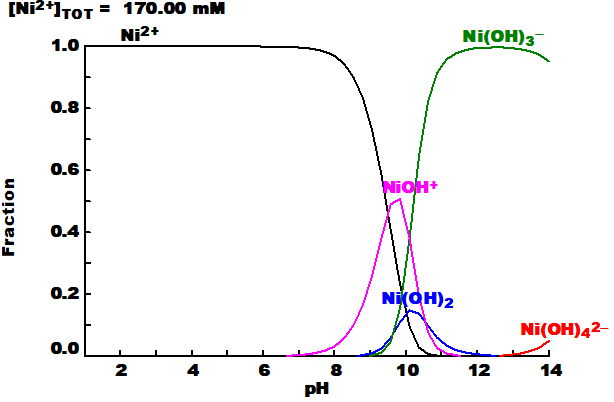


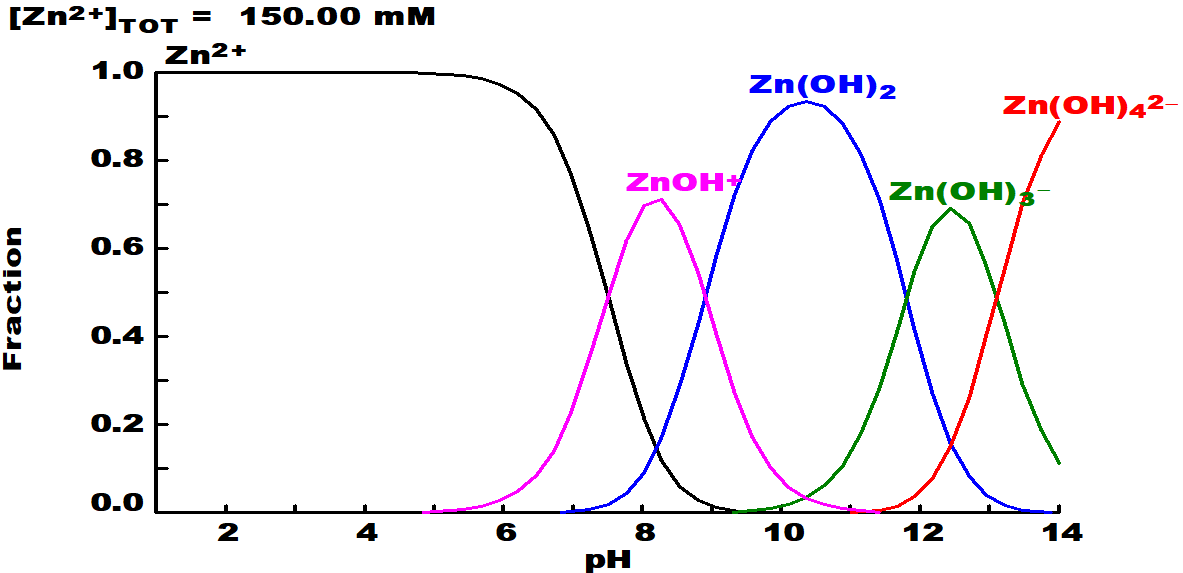


**Supplementary Figure 1.** Distribution diagram for M(II) species as a function of the solution pH at 25°C.

**(A)**

**(B)**

**(C)**

**Supplementary Figure 2.** Effect of pH on (A) Cu(II), (B) Zn(II), (C) Ni(II) adsorption on new polymeric microspheres (A=8, agitation speed 150 cycles/min, t=4 h).


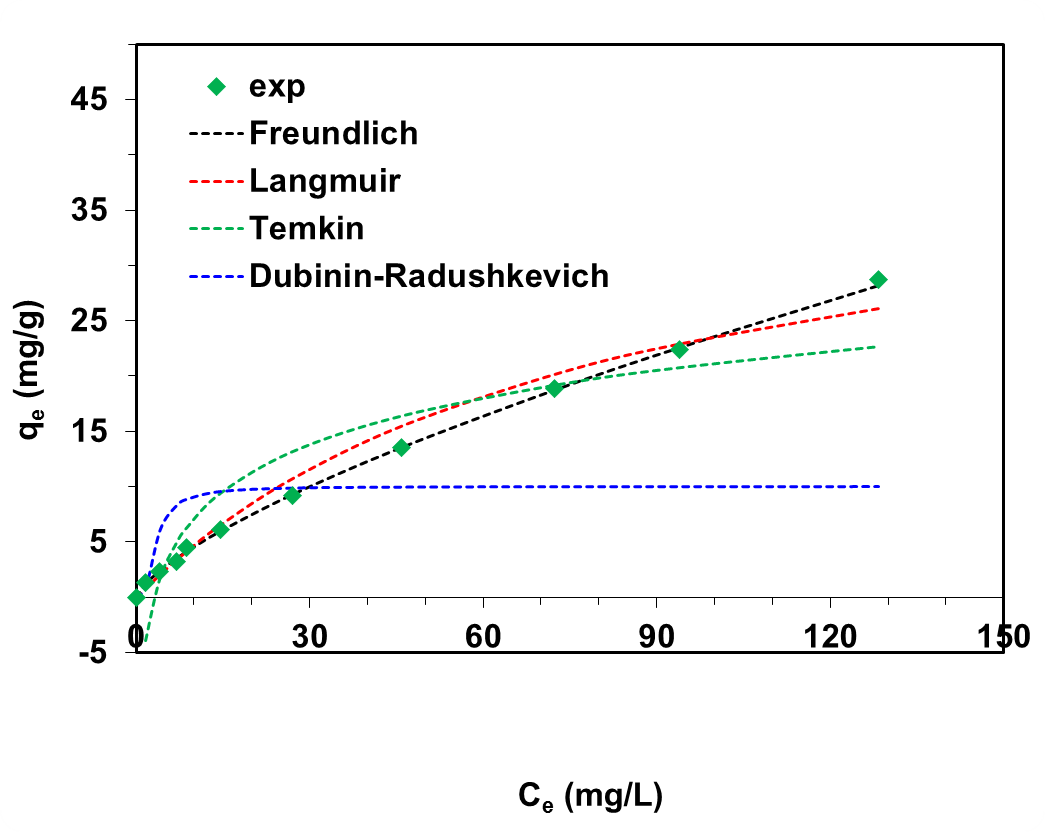


**EGDMA/VA+AG16**

**(A)**

**
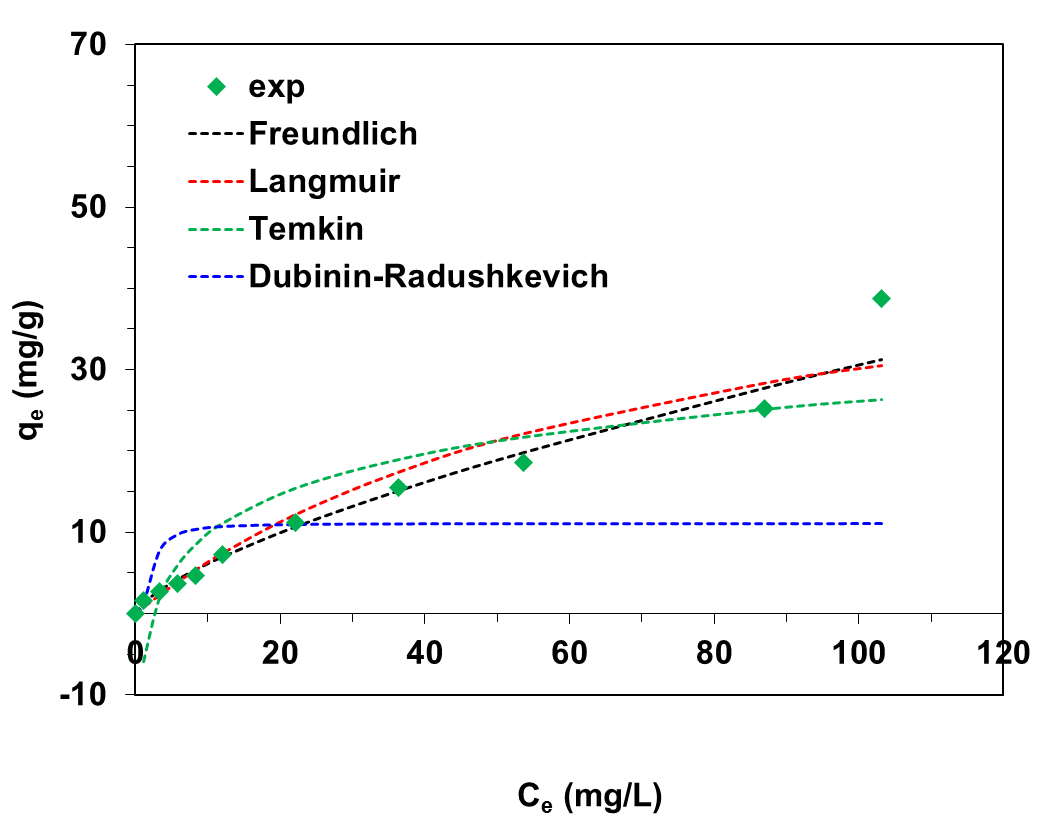
**

**EGDMA/VA-St/B+AG16**

**(B)**

**
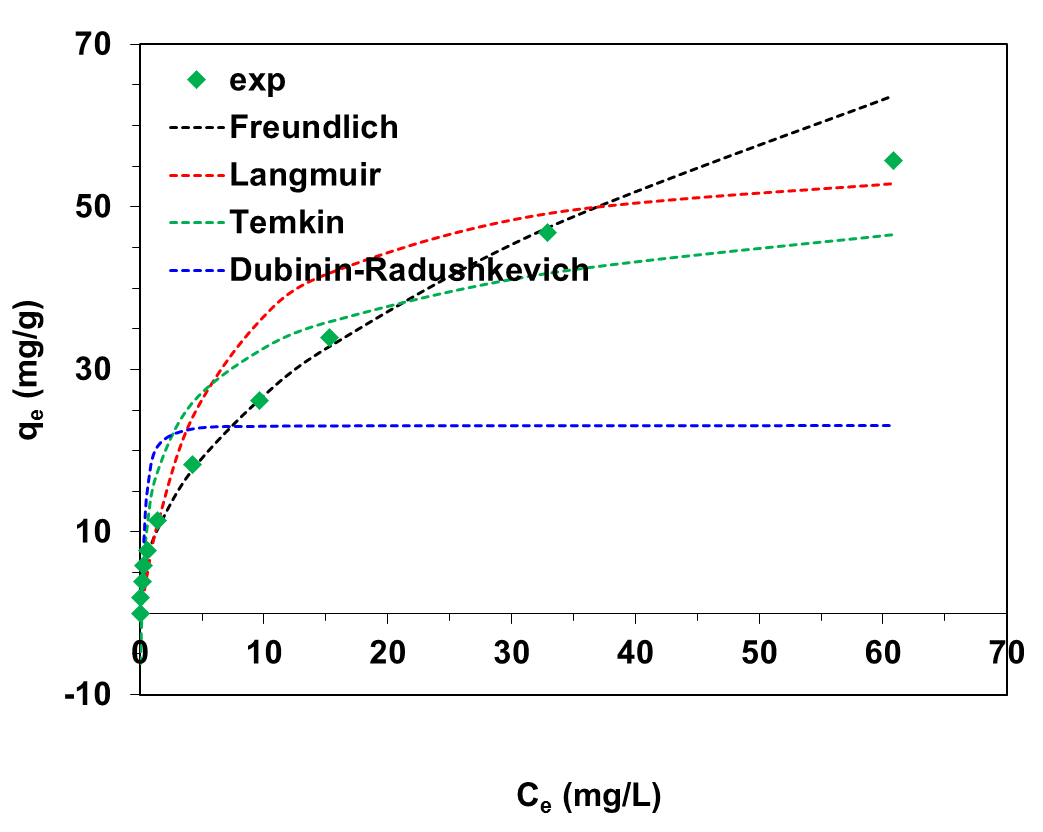
**

**EGDMA/VA-St/DiTDTA+AG16**

**(C)**

**
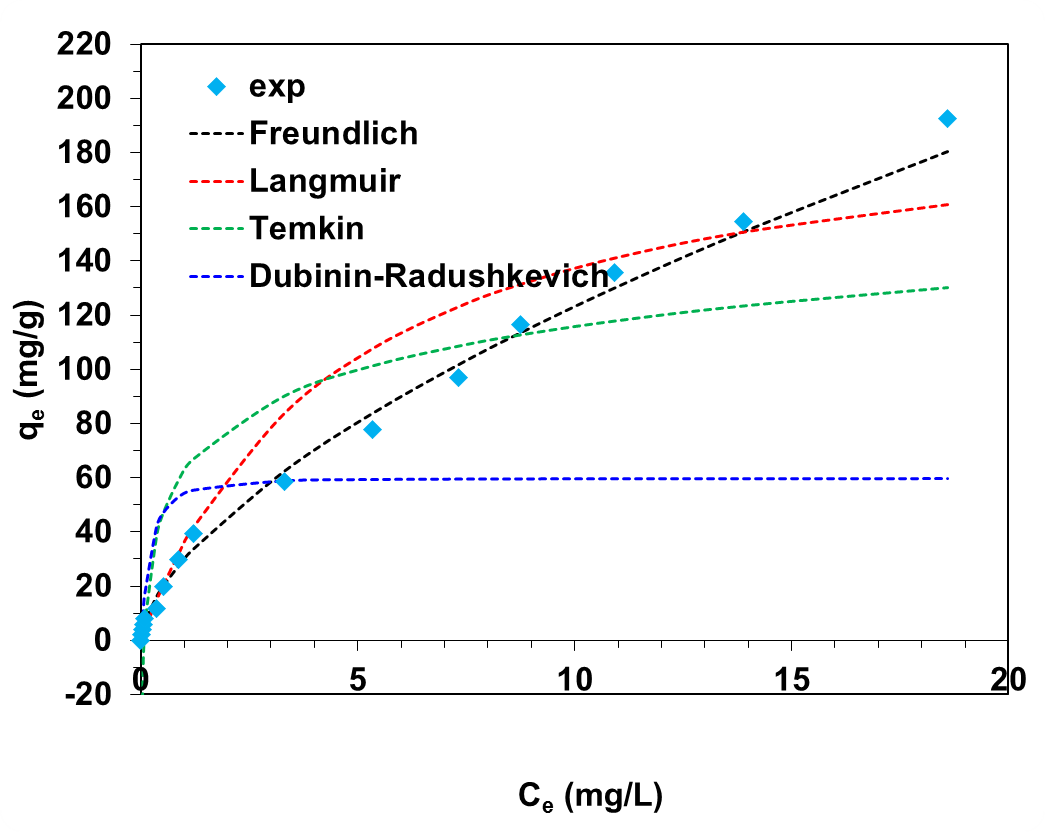
**

**EGDMA/VA+BB3**

**(D)**

**
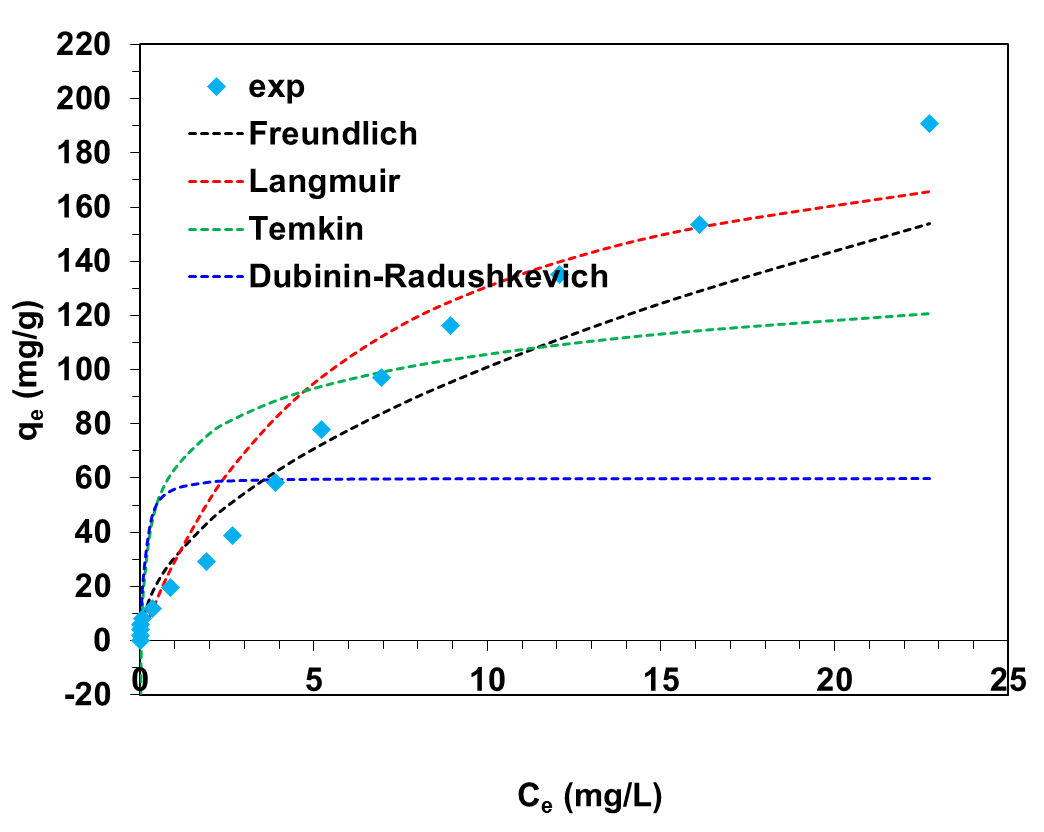
**

**EGDMA/VA-St/B+BB3**

**(E)**

**
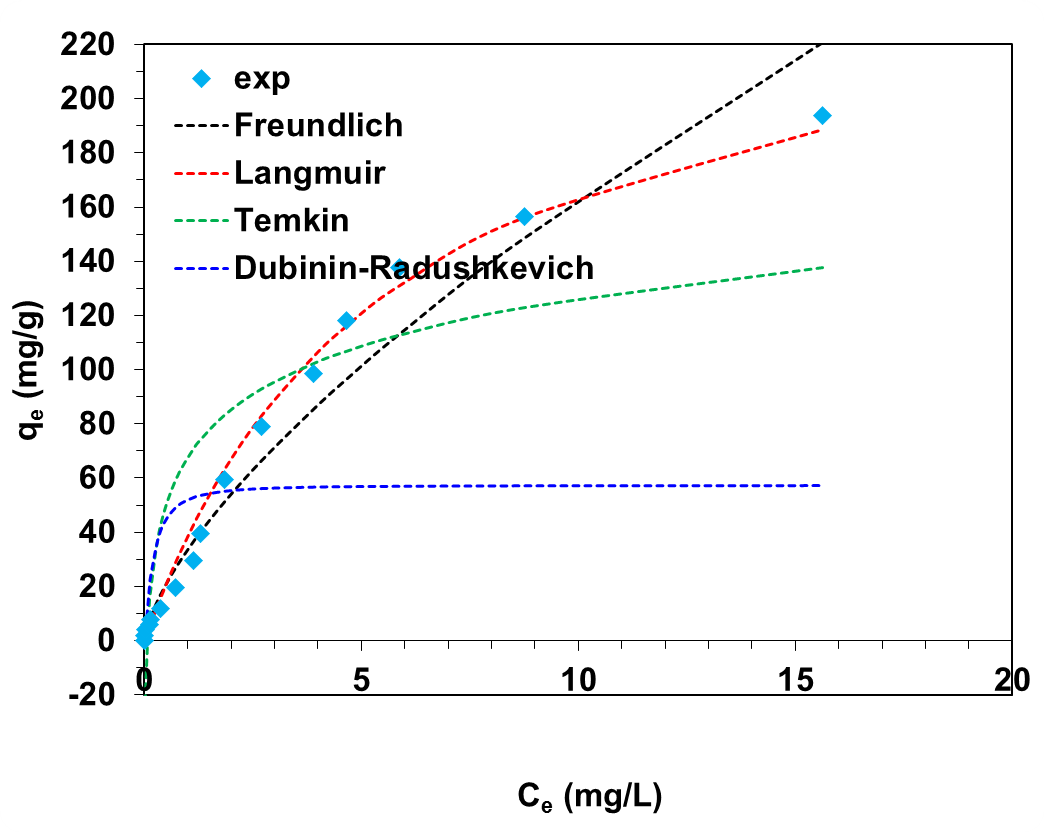
**

**EGDMA/VA-St/DiTDTA+BB3**

**(F)**

**
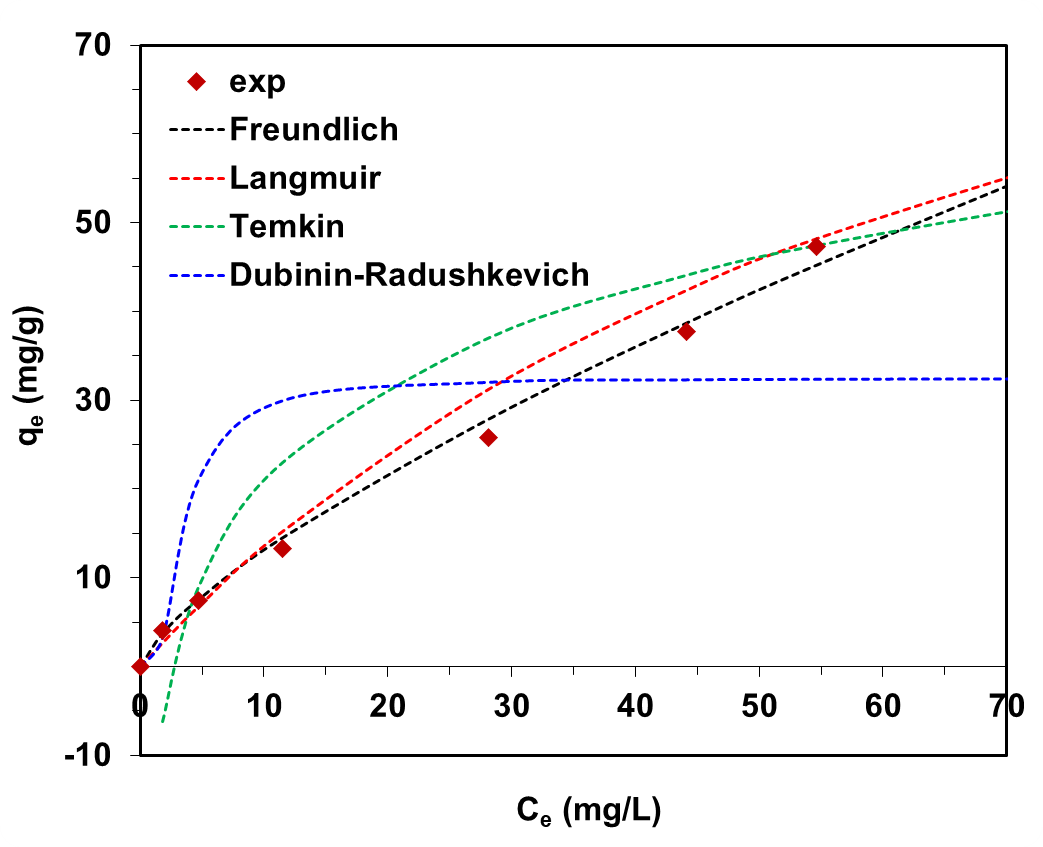
**

**EGDMA/VA+Cu(II)**

**(G)**

**
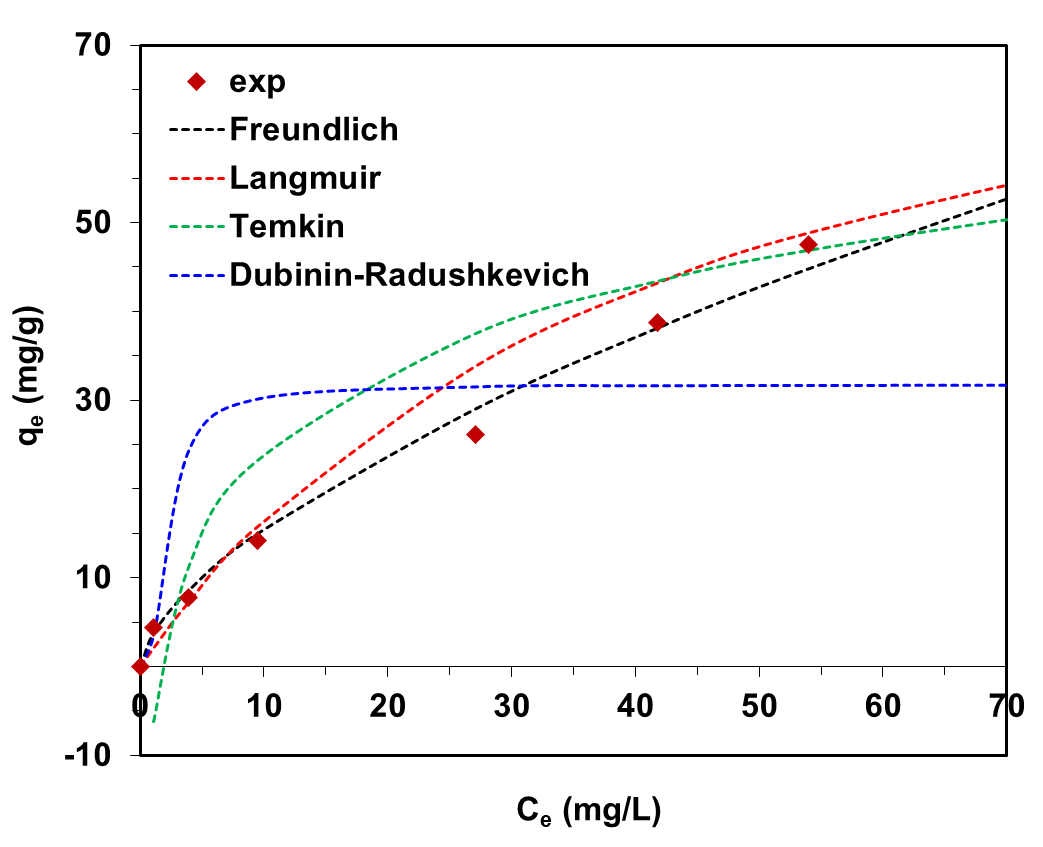
**

**EGDMA/VA-St/B+Cu(II)**

**(H)**

**
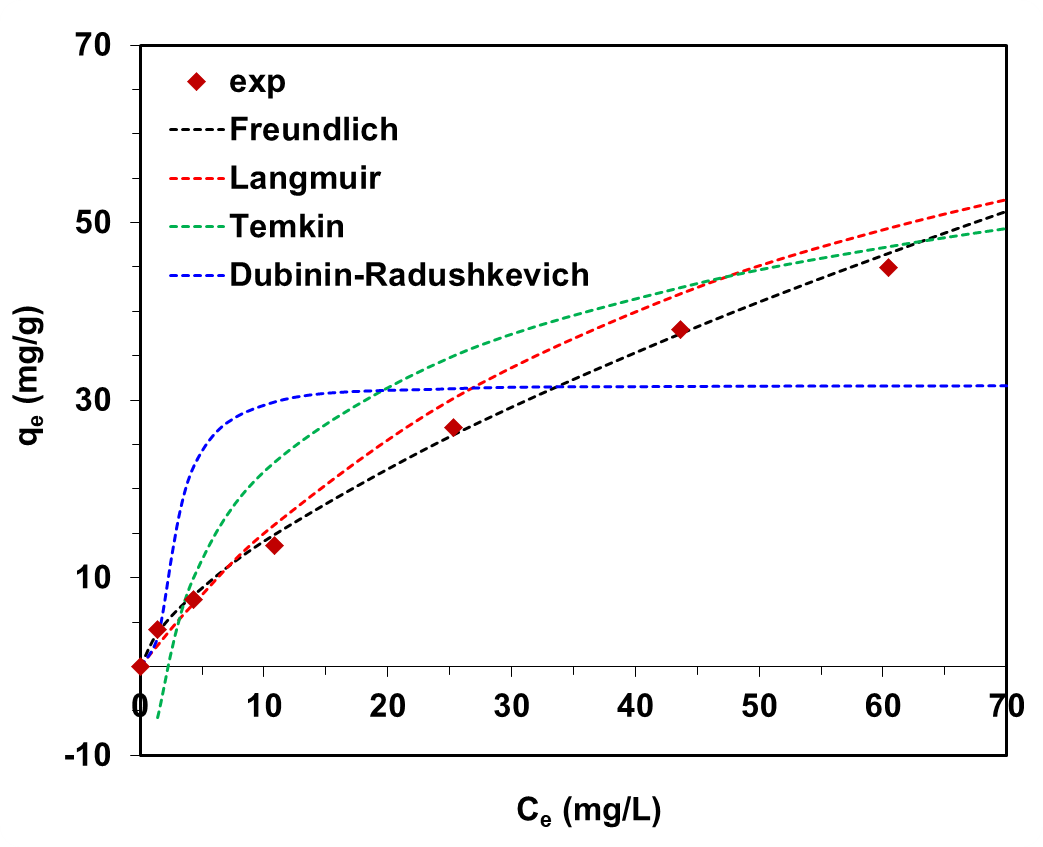
**

**EGDMA/VA-St/DiTDTA+Cu(II)**

**(I)**

**
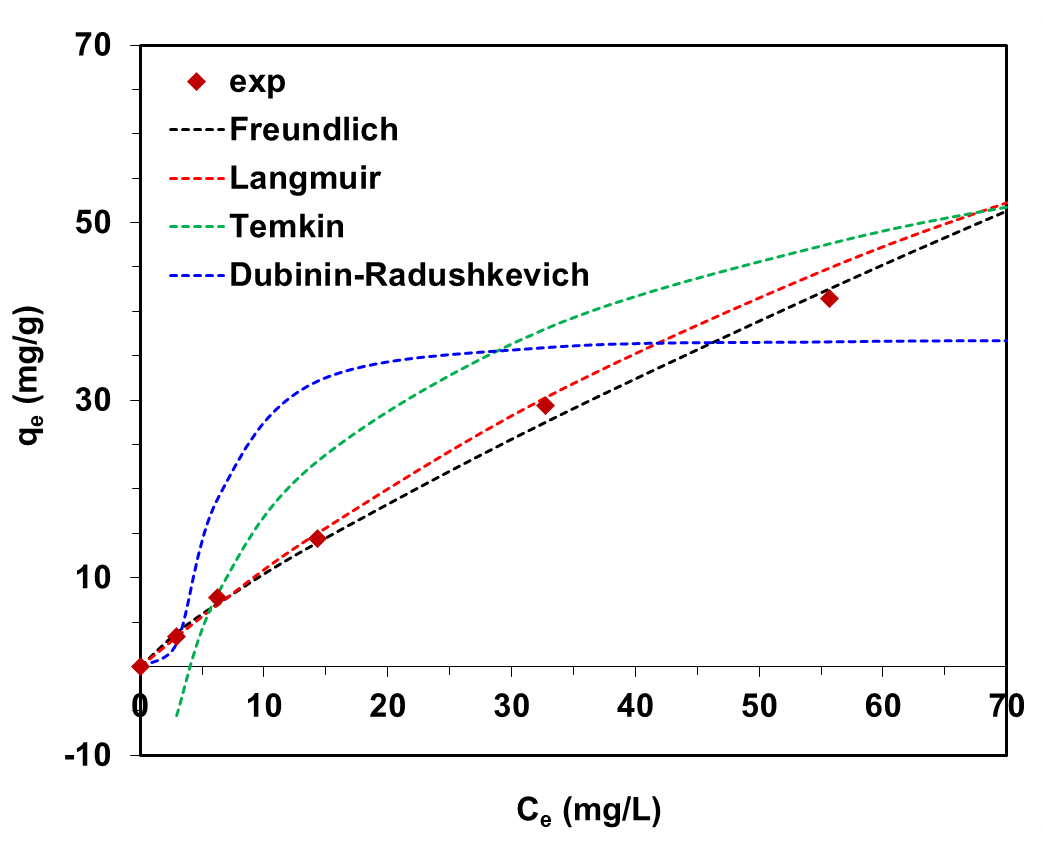
**

**EGDMA/VA+Ni(II) (II)**

**(J)**

**
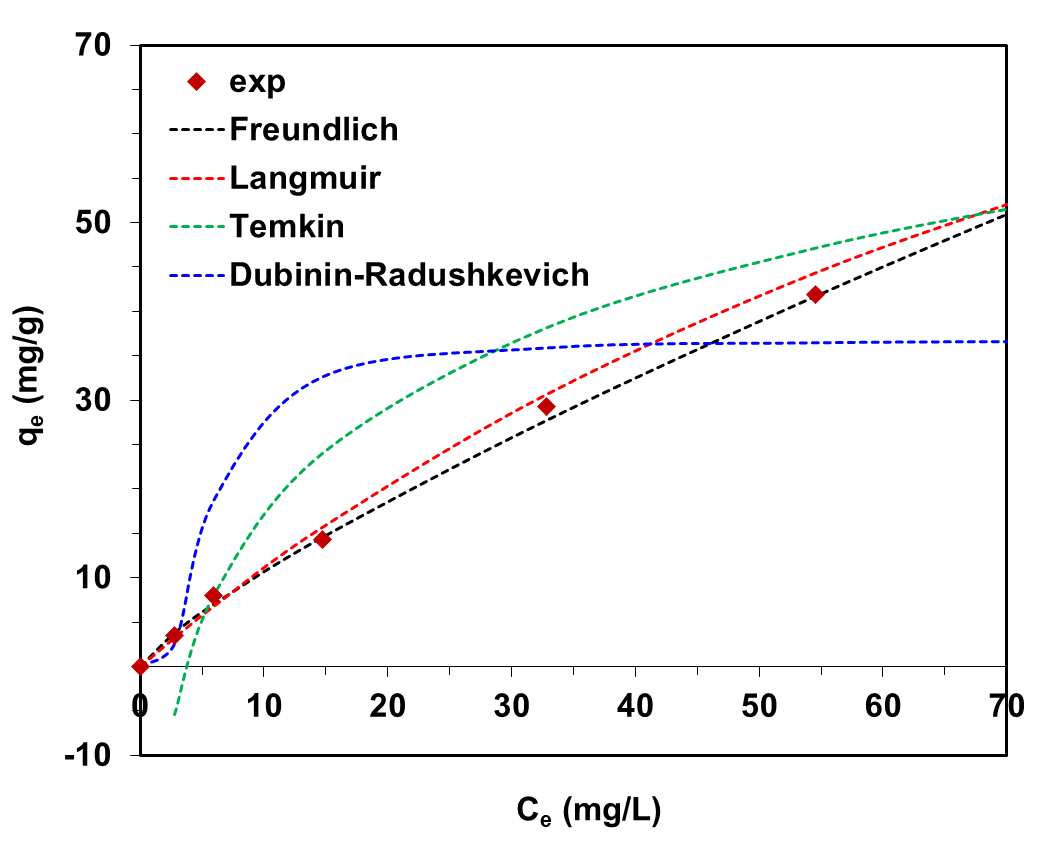
**

**EGDMA/VA-St/B+Ni(II)**

**(K)**

**
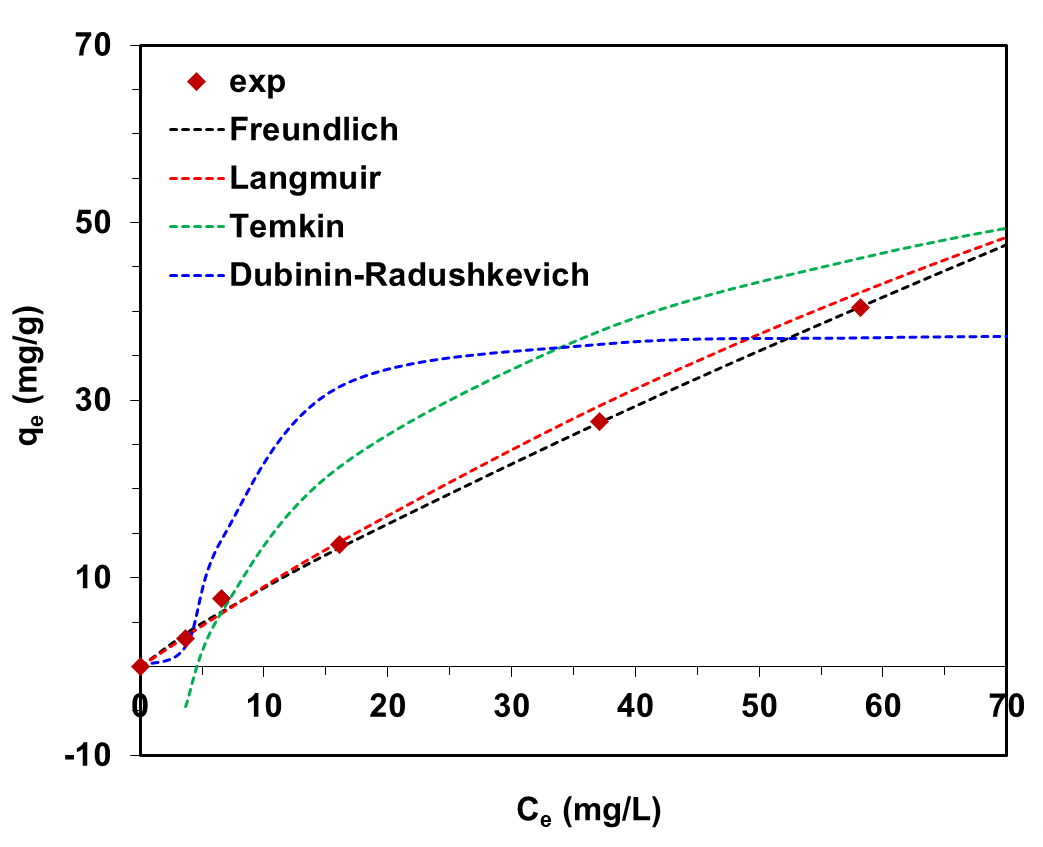
**

**EGDMA/VA-St/DiTDTA+Ni(II)**

**(L)**

**
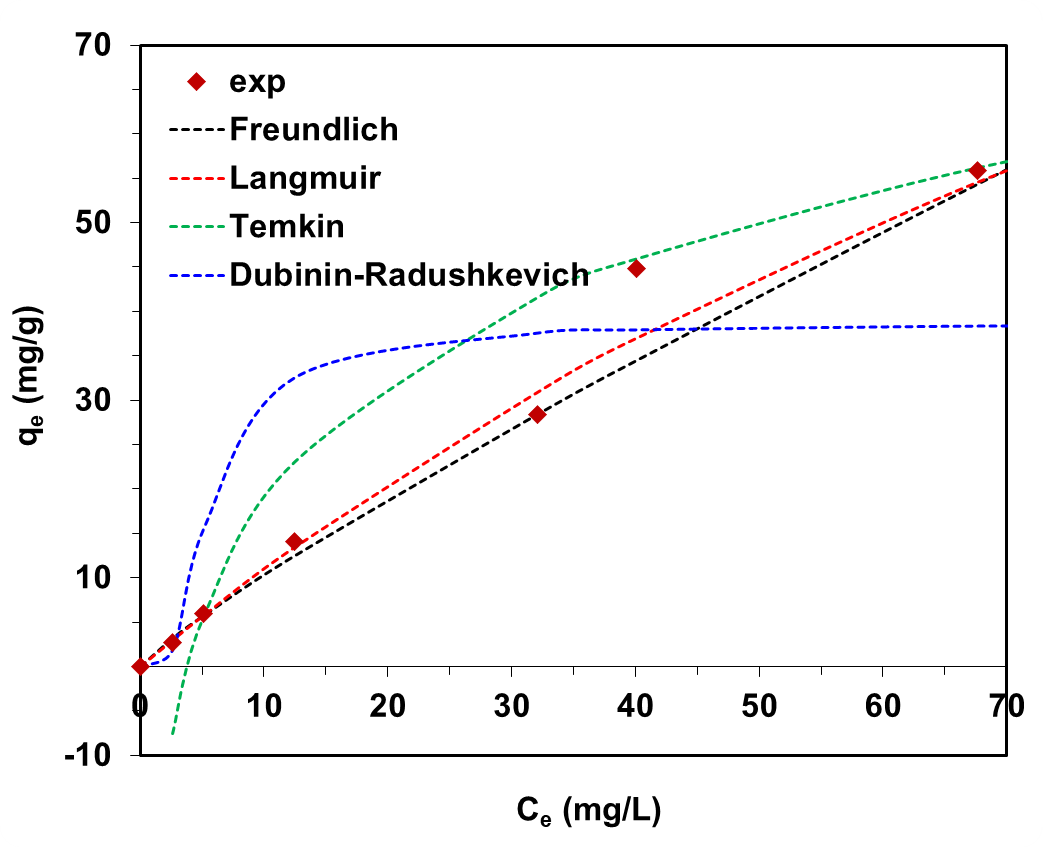
**

**EGDMA/VA+Zn(II) (II)**

**(M)**

**
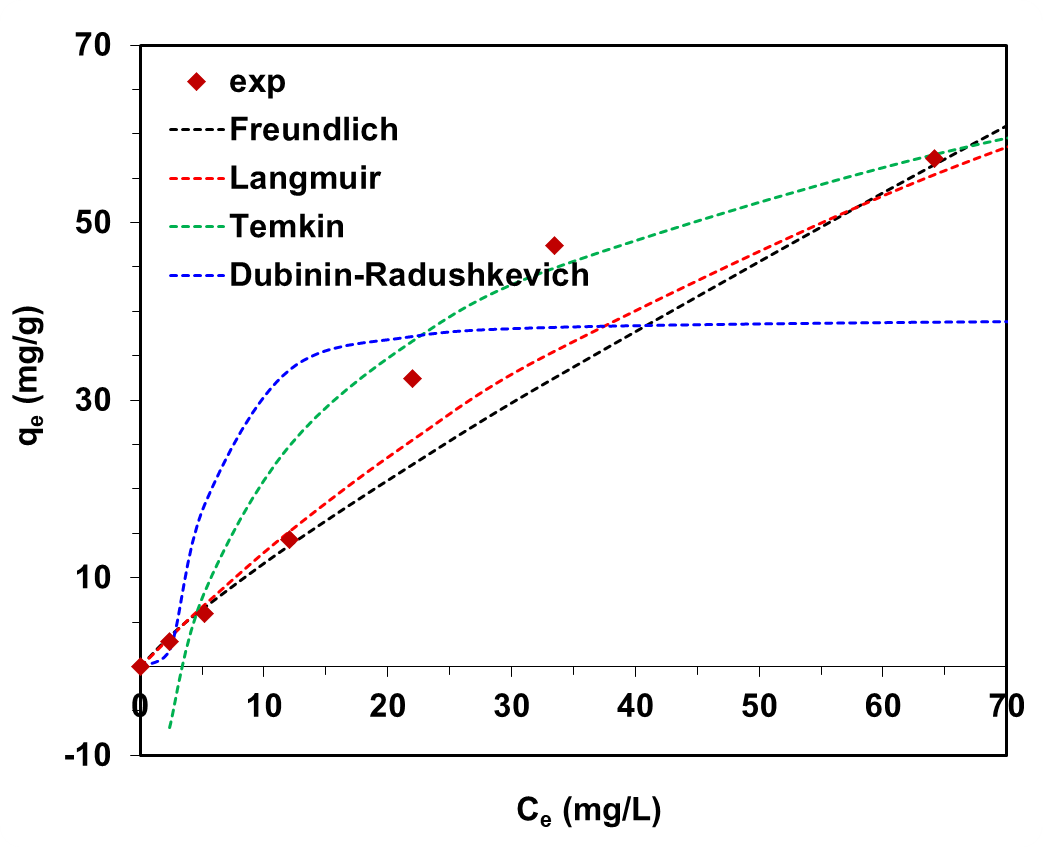
**

**EGDMA/VA-St/B+Zn(II)**

**(N)**

**
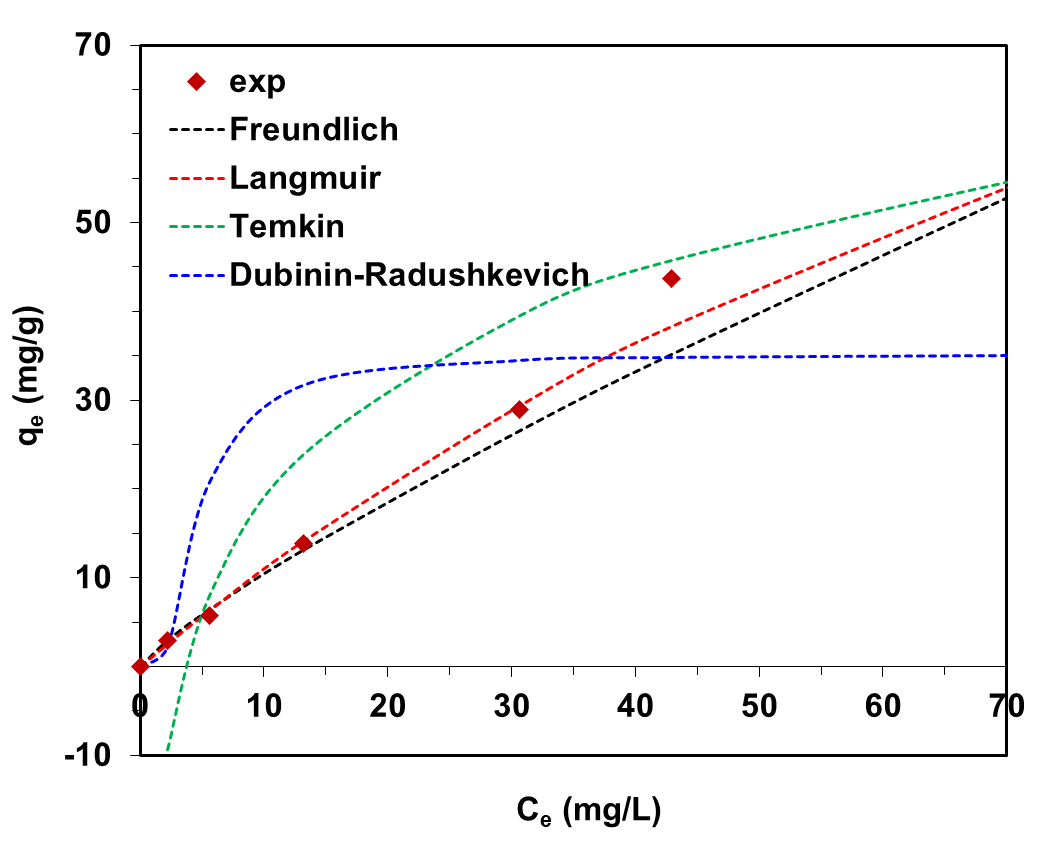
**

**EGDMA/VA-St/DiTDTA+Zn(II)**

**(O)**

**Supplementary Figure 3.** Fitting of equilibrium sorption data of dyes: AG16 (A-C), BB3 (D-F) and metal ions: Cu(II) (G-I), Ni(II) (J-L), Zn (M-O) to isotherm models in the systems dyes-polymeric adsorbent and heavy metal ions-polymeric adsorbent.

**(A)**

**(B)**

**(C)**

**(D)**

**Supplementary Figure 4.** The ATR/FT-IR spectra of EGDMA/VA (A,D), EGDMA/VA-St/B (B,D) and EGDMA/VA-St/DiTDTA (C,D) polymers after BB3, AG13 (A,B,C) and Cu(II) adsorption (D).

**
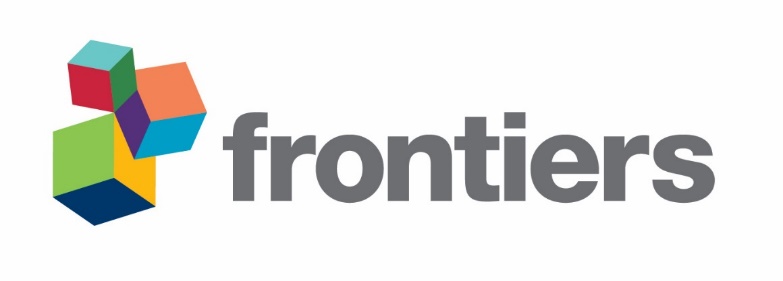
**
